# Conformational and mechanical stability of the isolated large subunit of membrane-bound [NiFe]-hydrogenase from *Cupriavidus necator*

**DOI:** 10.3389/fmicb.2022.1073315

**Published:** 2023-01-17

**Authors:** Jovan Dragelj, Chara Karafoulidi-Retsou, Sagie Katz, Oliver Lenz, Ingo Zebger, Giorgio Caserta, Sophie Sacquin-Mora, Maria Andrea Mroginski

**Affiliations:** ^1^Institut für Chemie, Technische Universität Berlin, Berlin, Germany; ^2^CNRS, UPR, Laboratoire de Biochimie Théorique, Université de Paris Cité, Paris, France; ^3^Institut de Biologie Physico-Chimique-Fondation Edmond de Rotschild, PSL Research University, Paris, France

**Keywords:** hydrogenase, molecular modelling, Gaussian accelerated molecular dynamics, rigidity profile, electrostatic potential, dipole moment, IR spectroscopy, size exclusion chromatography

## Abstract

Comprising at least a bipartite architecture, the large subunit of [NiFe]-hydrogenase harbors the catalytic nickel–iron site while the small subunit houses an array of electron-transferring Fe-S clusters. Recently, some [NiFe]-hydrogenase large subunits have been isolated showing an intact and redox active catalytic cofactor. In this computational study we have investigated one of these metalloproteins, namely the large subunit HoxG of the membrane-bound hydrogenase from *Cupriavidus necator* (*Cn*MBH), targeting its conformational and mechanical stability using molecular modelling and long all-atom Gaussian accelerated molecular dynamics (GaMD). Our simulations predict that isolated HoxG is stable in aqueous solution and preserves a large portion of its mechanical properties, but loses rigidity in regions around the active site, in contrast to the MBH heterodimer. Inspired by biochemical data showing dimerization of the HoxG protein and IR measurements revealing an increased stability of the [NiFe] cofactor in protein preparations with higher dimer content, corresponding simulations of homodimeric forms were also undertaken. While the monomeric subunit contains several flexible regions, our data predicts a regained rigidity in homodimer models. Furthermore, we computed the electrostatic properties of models obtained by enhanced sampling with GaMD, which displays a significant amount of positive charge at the protein surface, especially in solvent-exposed former dimer interfaces. These data offer novel insights on the way the [NiFe] core is protected from de-assembly and provide hints for enzyme anchoring to surfaces, which is essential information for further investigations on these minimal enzymes.

## Introduction

Dihydrogen (H_2_) is an important energy carrier that is extensively investigated in view of its potential interconnection with renewable energy sources. The development of affordable and efficient hydrogen-based technologies for energy storage and conversion (e.g., biofuel cells) took inspiration from the natural machinery involved in the H_2_ activation, namely the hydrogenase enzymes, which use earth-abundant transition metals for H_2_ production/oxidation (Ruff et al., 2018). Among them, [NiFe]-hydrogenases contain a heterobimetallic [NiFe] active site bound to the protein scaffold *via* four cysteine residues (Shafaat et al., 2013; Sickerman and Hu, 2019). Two of these cysteines bridge the Ni and Fe, the remaining two are bound terminally to the Ni while the coordination sphere of the Fe is completed by one CO and two CN-ligands (Rippers et al., 2012). Albeit the majority of [NiFe]-hydrogenases are strongly inhibited by molecular oxygen (O_2_), a few organisms have been shown to host O_2_tolerant enzymes enabling catalysis under oxic conditions. In our groups we have thoroughly investigated the membrane-bound hydrogenase (MBH) from *Cupriavidus necator* (*Cn*), formerly known as *Ralstonia eutropha* (Saggu et al., 2009; Goris et al., 2011) which belongs to the biotechnologically relevant subclass of O_2_-tolerant [NiFe]-hydrogenases (Lenz et al., 2015; Caserta et al., 2022b). The MBH catalytic architecture comprises the HoxG large subunit, which harbors the NiFe(CN)_2_(CO) catalytic site, and the small subunit HoxK containing three different electron-transferring [FeS]-clusters (Caserta et al., 2022b). Given the rare trait of being O_2_-tolerant, the MBH has been utilized for the development of various immobilization procedures on electrode surfaces to facilitate its applicability (Vincent et al., 2005; Utesch et al., 2013; Heidary et al., 2015; Harris et al., 2018). These procedures demand a detailed understanding of the structural, physical and chemical properties of the enzyme to ensure an efficient coupling between the biocatalyst and electrode surface (Utesch et al., 2013; Oteri et al., 2014b; Hitaishi, 2018).

More recently, [NiFe]-hydrogenase catalytic subunits have been successfully isolated independently of the small protein subunits (Hartmann et al., 2018; Kwon et al., 2018; Caserta et al., 2021, 2022a; Wang et al., 2021) and two of them from the model organism *C. necator* have been shown to host a redox-active O_2_-stable NiFe(CN)_2_(CO) active site (Caserta et al., 2021, 2022a). These are the HoxC subunit from the regulatory hydrogenase (RH) and the HoxG from MBH, and both could activate molecular hydrogen to a little extent (Hartmann et al., 2018; Caserta et al., 2020a). While several crystal structures of heterodimeric [NiFe]-hydrogenases (*Cn*MBH included) have been reported (Fontecilla-camps et al., 2007; Fritsch et al., 2011; Frielingsdorf et al., 2014) and detailed information on the substrate/product routes are available (Kalms et al., 2016, 2018), the structure of an isolated catalytic subunit of such [NiFe]-hydrogenase that solely harbors the active [NiFe] site, has not been experimentally determined. Additionally, despite spectroscopy having provided detailed insights on the electronic and ligands arrangements of the [NiFe] cofactor (Caserta et al., 2020a, 2021, 2022a), little is known about the protein arrangement and the conformational changes of the catalytic subunit upon removal of the small protein subunit (Kwon et al., 2018). Several computational studies have been already performed on the heterodimeric hydrogenases [for data on *Cn*MBH see, e.g. (Teixeira et al., 2006; Utesch et al., 2013; Oteri et al., 2014a,b; Heidary et al., 2015; Kalms et al., 2018; Albareda et al., 2019)], however, only very few of them have focused on the isolated hydrogenase catalytic subunits (Albareda et al., 2019). In this work we focus on the conformational stability of the MBH catalytic subunit, the HoxG protein, which was investigated using molecular modeling and extensive Gaussian accelerated molecular dynamics (GaMD) (Miao et al., 2015). Accelerated MD is a computational approach used to enhance sampling of the conformational space of large molecular systems by artificially decreasing energy barriers of the potential energy surface that surpass a certain energy threshold. This allows the population of conformational states, which are inaccessible with conventional classical MD (cMD) simulations in the same time span (Hamelberg et al., 2004). In GaMD, the boost potential follows a near-Gaussian distribution that ensures simultaneous energetic noise reduction and free energy computations (Miao et al., 2015). These approaches can be used for investigating structural, mechanical, and electrostatic properties of large (bio)molecular systems in complex heterogeneous environments such as proteins attached to membranes (Tillmann, 2018) or immobilized on electrodes (Utesch et al., 2013; Heidary et al., 2015). By combining GaMD with Coarse-grained Brownian Dynamics (BD) simulations (Sacquin-Mora, 2014, 2016, 2018), we targeted the conformational space and predicted the mechanical properties as well as the structural stability of HoxG protein. Our computational work shows the consequences of reducing a whole enzyme to its catalytic unit. Finally, supported by biochemical and infrared spectroscopic data, we revealed a direct correlation between the HoxG oligomerization states and their influence on the active site stability. The atomistic information gained within this combined computational/experimental approach is essential not only for understanding the consequences of reducing an enzyme architecture to its catalytic unit but also for rationally designing new “smaller” constructs with comparable stability and, in turn, biological activity to the native system.

## Materials and methods

In the following, models of the large subunit HoxG will be labelled as follows: HoxG_m_: thermodynamically equilibrated monomeric form, HoxG_d_: thermodynamically equilibrated homodimeric form, HoxG_c_: isolated HoxG in crystallographic arrangement; HoxG_MBH_: HoxG complexed with HoxK (small subunit) in crystallographic arrangement.

### Structure preparation and modelling of the large subunit of MBH (HoxG_m_)

**Figure 1:**
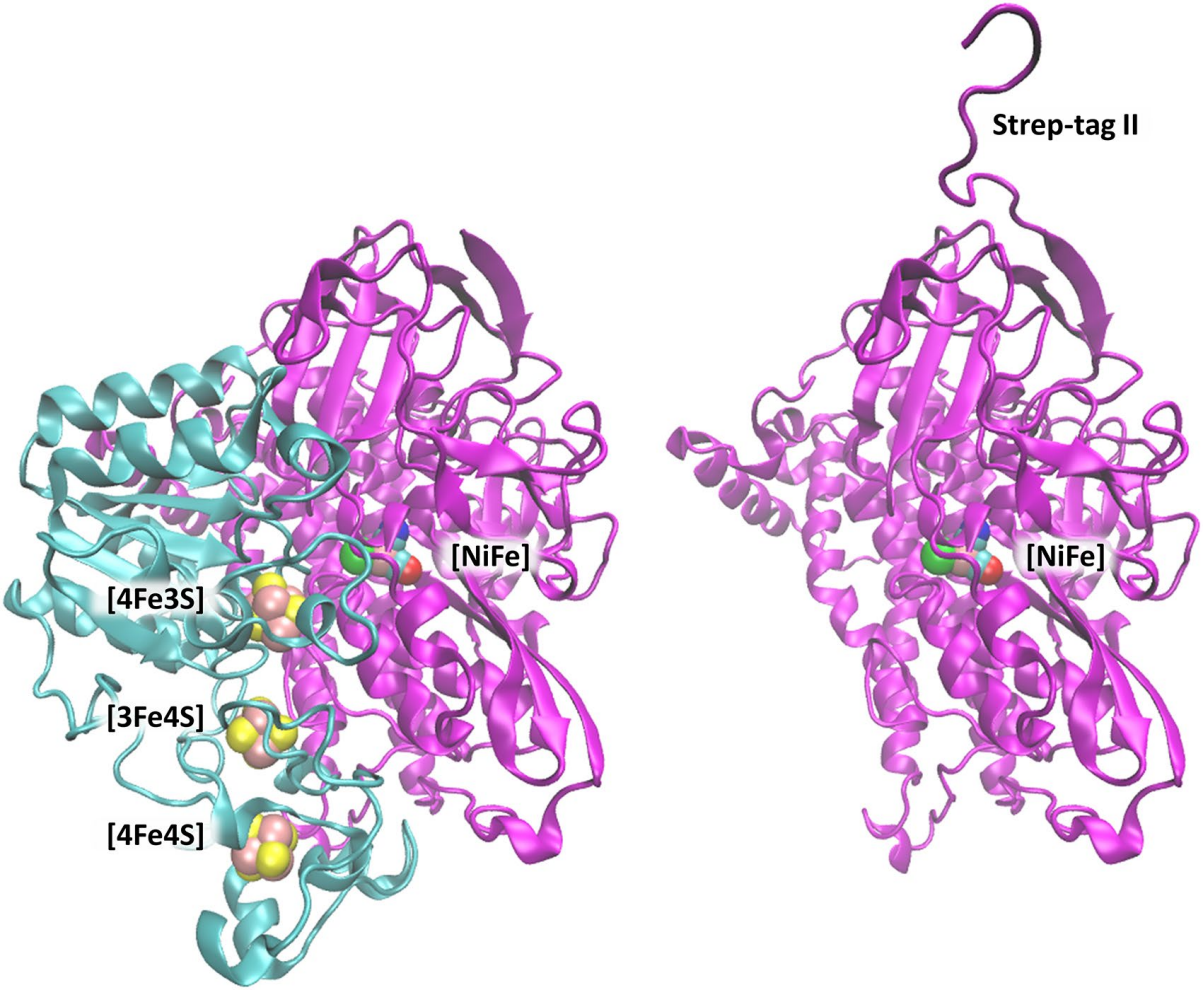
[NiFe]-hydrogenase from *C. necator* (PDB code: 3RGW (Fritsch et al., 2011)). **Left**: Heterodimer consisting of the small subunit (HoxK, colored cyan) and large subunit (HoxG, colored magenta). **Right**: Isolated large subunit (HoxG) with its Strep-tag II sequence located at the N terminus (Caserta et al., 2022a). [NiFe]-active site and [FeS]-clusters are shown as spheres and depicted with the following color code: Fe in pink, Ni in green, S (from Cys) in yellow, CN-and CO groups, cyan-blue and cyan-red, respectively.

The X-ray crystal structure of the heterodimeric MBH from *C. necator* (MBH; PDB code: 3RGW (Fritsch et al., 2011; Figure 1) was used to extract coordinates of the large subunit (HoxG) as a starting model. During the natural maturation of MBH, the HoxG subunit forms a heterodimer with HoxK only after insertion of the [NiFe] cofactor and cleavage of its C-terminal tail (Hartmann et al., 2018, 2020; Caserta et al., 2022a).

Therefore, this C-terminal peptide is not found in the heterodimeric crystal structure, and it is not present in our model targeting a fully mature HoxG subunit.

The HoxG protein is equipped with a Strep-tag II at its N terminus (Caserta et al., 2022a); therefore, we have used CHARMM software (Brooks et al., 2009) and CHARMM 36 Force-Field (MacKerell, 1998; Best et al., 2012) to model the affinity tag. After prediction using i-Tasser (Zhang, 2008; Roy et al., 2010; Yang et al., 2014), the Strep-tag II sequence has been modelled in a random coil conformation, followed by a geometry optimization in the dielectric medium (ε = 4) for 4,000 steps. Additionally, a short geometry optimization of Strep-tag II while keeping the coordinates of HoxG fixed was done with NAMD (Phillips et al., 2005) in vacuum for 10 ns. The protonation pattern has been computed with Karlsberg2^+^ software (Rabenstein and Knapp, 2001; Kieseritzky and Knapp, 2008; Meyer and Knapp, 2015) at pH 7.0 and applied to titratable groups in all MD simulations (see SI; Supplementary Table S1). The resulting structure with modelled cofactors has been used as a basis for all further MD simulations, both conventional (cMD) and Gaussian accelerated (GaMD; Miao et al., 2015). Amino-acid numbering has been kept following that of the MBH crystal structure. Where necessary, the amino acid numbering of the Strep-tag II sequence has been included with the prefix “tag.”

### Atomic partial charges and modelling of the [NiFe]-active site

The [NiFe] active site of MBH has been trapped in several redox states of the catalytic cycle (Sickerman and Hu, 2019; Apfel et al., 2020). For simplicity, we have chosen an EPR silent state, the Ni_a_-S state (Ilina et al., 2019; Sickerman and Hu, 2019), which represents the presumed state accepting and releasing H_2_. In the Ni_a_-S state, both Ni and Fe are in a + 2 redox state, coordinated to four cysteine residues. The atomic partial charges have been taken from early works on similar enzymes (Teixeira et al., 2006), where electrostatic potential was computed quantumchemically with Gaussian (Frisch, 2016) and fitted with RESP (Cornell et al., 1993). During preparatory steps and MD simulations, the [NiFe] active site has been kept rigid with coordinates as in the crystal structure but with corrected ligand orientation [Rippers et al., 2012; PDB code: 3RGW (Fritsch et al., 2011)] by applying conformational restraints on bonds (force constant of 500 kcal), angles (force constant of 100 kcal) and dihedral angles (barrier of 1.0 kcal) with NAMD (Phillips et al., 2005), as in previous applications (Utesch et al., 2013; Heidary et al., 2015). Considering that the main goal of this study is to investigate the global dynamic properties of the large subunit, constraints on the [NiFe] active site will have little to no influence.

### Molecular dynamics (MD) simulations

The prepared structure of the large subunit with the N-terminal Strep-tag II and crystal water molecules were solvated using a TIP3P (Jorgensen et al., 1983) water box with periodic boundary conditions *via* a PSFGEN VMD plugin (Humphrey et al., 1996) with an addition of NaCl ions (150 mM). A water box with slightly larger dimensions 102 Å x 121 Å x 93 Å was used to accommodate conformational changes stemming from Strep-tag II flexibility or other protein regions. All MD simulations (conventional and Gaussian accelerated) were performed with software NAMD (Phillips et al., 2005) with 2 fs time step using SHAKE (Ryckaert et al., 1977) fixing bond lengths of hydrogen atoms, Langevin dynamics at 300 K with small friction constant of β = 1 ps^−1^ avoiding slowing down of dynamics (Blumhagen et al., 1996) and particle-mesh Ewald method (Darden et al., 1993) for electrostatic interactions.

The solvated model system described above was energy minimized, then heated to 300 K for 20 ps while restraining the positions of all protein heavy atoms. During the 60 ps-long preequilibration step, the conformational restraints on the protein atoms were gradually lifted except for the atoms constituting the [NiFe] active site including the three inorganic ligands and the side chains atoms (up to Cβ) of the coordinating cysteine residues. Finally, the entire system was thermally equilibrated for 45 ns *via* cMD. The preparatory steps before GaMD included a 2 ns extension of the cMD in order to collect potential statistics for the estimation of the GaMD (double boost) acceleration parameters and a 2 ns GaMD equilibration considering the previously calculated boost potential (Miao et al., 2015). GaMDs were run for 250 ns. During these simulations the upper limits of the standard deviation of the total boost potential and the dihedral boost potential were set to 5 kcal/mol. To enlarge the statistical sampling, both cMD and GaMD were repeated twice. Further in text, these simulations are referred to as simulation 1 and simulation 2.

### Electrostatic energy and pK_A_ computations

The initial protonation pattern (Supplementary Table S1; SI) was determined based on the initial model [HoxG from the crystal structure 3RGW (Fritsch et al., 2011) with modelled N-terminal Strep-tag II as a random coil, Figure 1], by computing pK_A_ values with Karlsberg2^+^ (KB2^+^; Rabenstein and Knapp, 2001; Kieseritzky and Knapp, 2008; Meyer and Knapp, 2015), as in previous applications (Wolf et al., 2020; Dragelj et al., 2021a,b; Batebi et al., 2018). The pK_A_ values have been computed in the range from −10.00 to 20.00, not only for the starting model but also for the structures from GaMD trajectories obtained every 2 ns. The conformational stability of predicted structures was evaluated considering conformational energies from the time frames of GaMDs. Conformational energies were computed by solving the linearized PoissonBoltzmann (LPB) equation with the program “Adaptive Poisson-Boltzmann Solver” (APBS) (Baker et al., 2001). Water molecules and electrolytes were removed and replaced by an implicit ion concentration of 150 mM. The region out of the solvent accessible surface area was treated as a dielectric continuum with ε = 80 and protein volume was treated as a dielectric continuum with ε = 4. These conformational energies were computed using a grid resolution of 0.3 Å for frames taken every 4 ns from the resulting trajectories.

### Computation of mechanical properties *via* coarse-grain simulations

Coarse-grained Brownian Dynamics (BD) simulations were run using the ProPHet (Probing Protein Heterogeneity, available online at https://bioserv.rpbs.univ-paris-diderot.fr/services/ProPHet/) program (Sacquin-Mora, 2014, 2016, 2018). In this approach, the protein is represented using an elastic network model (ENM). Unlike most common coarse-grained models where each residue is described by a single pseudo atom (Tozzini, 2005) ProPHet uses a more detailed representation (Zacharias, 2003) that involves up to 3 pseudo atoms per residue and enables different amino acids to be distinguished. Pseudo atoms closer than the cutoff parameter R_c_ = 9 Å are joined by Gaussian springs which all have identical spring constants of γ_struct_ = 0. 42 N.m^−1^ (0.6 kcal.mol^-1.^Å^−2^). The springs are taken to be relaxed for the initial conformation of the protein. The simulations use an implicit solvent representation *via* the diffusion and random displacement terms in the equation of motion, (Ermak and McCammon, 1978) and hydrodynamic interactions are included through the diffusion tensor (Pastor et al., 1988).

Mechanical properties are obtained from 200,000 BD steps at an interval of 10 fs and a temperature of 300 K. The simulations lead to deformations of roughly 1.5 Å root-mean-square deviation with respect to the protein starting conformation (which by construction corresponds to the system’s equilibrium state). The trajectories are analyzed in terms of the fluctuations of the mean distance between each pseudo atom belonging to a given amino acid and the pseudo atoms belonging to the remaining protein residues. The inverse of these fluctuations yields an effective force constant *k_i_* describing the ease of moving a pseudo atom with respect to the overall protein structure:


$$k_{i}=\frac{3k_{B}T}{\langle {\left(d_{i}-\langle d_{i}\rangle \right)}^{2}\rangle },$$

where 〈〉 denotes an average taken over the whole simulation and *d_i_ =* 〈*d_ij_*〉j* is the average distance from particle *i* to the other particles *j* in the protein (the sum over j* implies the exclusion of the pseudo atoms belonging to residue *i*). The distance between the C_α_ pseudo atom of residue *i* and the C_α_ pseudo atoms of the adjacent residues *i-1* and *i + 1* are excluded since the corresponding distances are virtually constant. The force constant for each residue is the average of the force constants for all its constituent pseudo atoms *i*. We will use the term *rigidity profile* to describe the ordered set of force constants for all the residues of the protein.

### Modelling and MD simulations of the homodimeric large subunit of MBH (HoxG_d_)

**Figure 2:**
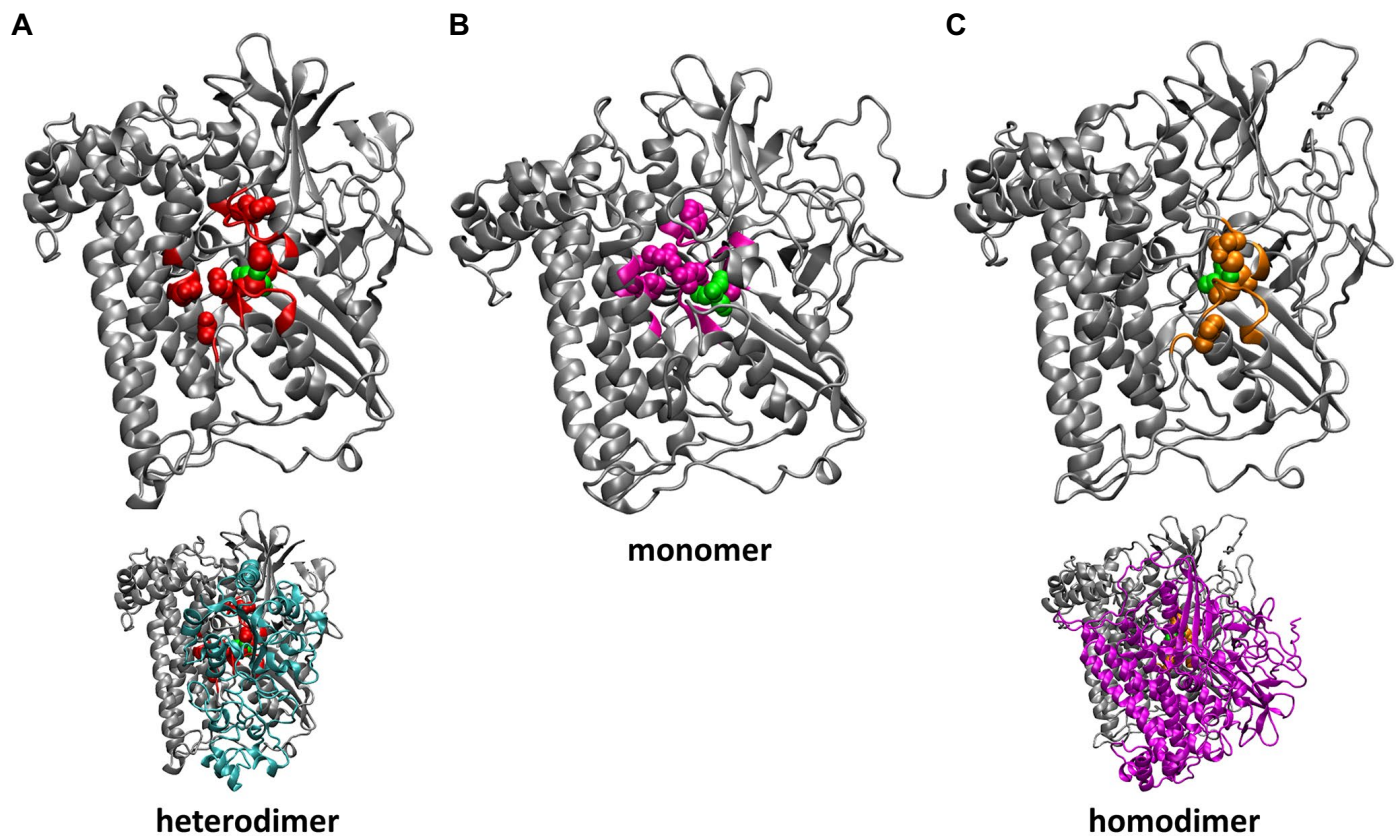
Residues of HoxG with highest rigidity constants (100 kcal·mol^−1^·Å^−2^): in the crystal structure of *Cn*MBH, HoxG_MBH_ (**A**, red), in a HoxG_m_ structure taken from GaMD simulation 2 at 200 ns (**B**, magenta) and in one monomer of the HoxG_d_ structure taken from GaMD simulation at 150 ns (**C**, gold). The structures of the entire *Cn*MBH heterodimer and the entire HoxG_d_ are also displayed (**A**,**C**, bottom). The [NiFe] active site is shown in green. The protein backbone of residues with force constants less than 100 kcal·mol^−1^·Å^−2^ is shown in grey.

Despite the experimental evidence that isolated preHoxG (i.e., a HoxG protein precursor equipped with an [NiFe] active site but still containing its C-terminal extension tail) can be found in different oligomerization states with a prevalent homodimeric fraction, no detailed structural information was available (Hartmann et al., 2018). The three-dimensional structure of the HoxG homodimer was constructed using the cartesian coordinates of the monomeric structure predicted with the lowest conformational energy during 20 ns of GaMD simulation. We used SymmDock Webserver (Schneidman-Duhovny et al., 2005a,b) to investigate the relative orientation of two HoxG_m_ subunits. The SymmDock algorithm predicts protein complexes by geometry-based rigid docking, whereby the scoring function considers both geometric fit and atomic desolvation energy of resulting constructs. In our protocol we requested the docking of two isolated HoxG units without distance constraints, nor the prior definition of a binding interface. Interestingly, out of the resulting top 10 suggested complexes more than half of them form dimer structures in which both HoxG monomers interact with each other *via* the HoxG-HoxK interface of the functional MBH heterodimer as seen in Figure 2. For simplicity, we have chosen a HoxG_d_ model with the highest overall score as a starting model, as the prediction of reliable dimer poses can be computationally very demanding, and it is not the main objective of this work. The HoxG_d_ model was prepared for MD simulations following the same protocols and conditions as described above for the HoxG_m_ with an unchanged protonation pattern [determined by Karlsberg2^+^ software (Rabenstein and Knapp, 2001; Kieseritzky and Knapp, 2008; Meyer and Knapp, 2015). HoxG_d_ was solvated in a water box with dimensions of 121 Å x 114 Å x 114 Å. After 45 ns thermal equilibration at 300 K with cMD, boost parameters for GaMD were estimated for a 150 ns long GaMD of the HoxG_d_ model.

### Size exclusion chromatography

For the size exclusion chromatography experiments, the as-purified HoxG protein was investigated in various concentration ranges (0.5, 2.5, 12.5 and 60 mg/ml) in 50 mM K_i_PO_4_, 150 mM NaCl pH 7.4 (purification buffer). Measurements were run on an ÄKTA pure 25 using a Superdex 200 Increase 10/300 GL (Cytiva) column equilibrated with the purification buffer at 4 °C. A calibration curve was made by measuring six protein standards with known molecular weights between 12 and 670 kDa: Thyroglobulin (669 kDa, 9.34 ml), Apoferritin (443 kDa, 10.49 ml), β-Amylase (200 kDa, 11.8 ml), Bovine serum albumin (66 kDa, 14.1 ml), Carbonic anhydrase (29 kDa, 16.8 ml) and cytochrome C (12.3 kDa, 18.24 ml). Additionally, HoxC (the large subunit of the regulatory hydrogenase from *C. necator*) was included in the calibration series as its oligomerization profile was recently elucidated (Caserta et al., 2020a).

### IR spectroscopy

HoxG protein solutions were transferred into a homemade, gas-tight, and temperaturecontrolled (10°C) transmission cell equipped with two sandwiched CaF_2_ windows separated by a Teflon spacer with an optical pathlength of 50 μm. Spectra with a resolution of 2 cm^−1^ were recorded on a Tensor 27 Fourier-Transform spectrometer (Bruker) equipped with an MCT (liquid nitrogen-cooled mercury-cadmium-telluride) detector. The cell compartment was purged with dried air. For a single spectrum 200 individual scans were averaged. A buffer spectrum was used as reference for calculating the corresponding absorbance spectra. OPUS software version 7.5 from Bruker was used for data analysis. For the cofactor-stability experiments, IR spectra of as-isolated HoxG protein solution 35 mg/ml (500 μM) and a threefold higher concentrated sample were recorded consecutively for 7 h.

## Results

### Conformation of the isolated large subunit HoxG_m_

The structures obtained from Gaussian accelerated molecular dynamics simulations were analyzed in order to get a deeper insight into the dynamics and stability of the HoxG_m_ subunit. Special attention has been given to the quaternary structure, as well as the preservation of secondary structure elements. Root-meansquare deviation (RMSD), root-mean-square fluctuation (RMSF) of backbone atoms of protein, electrostatic conformational energy, and rigidity profile of the structure were computed.

**Figure 3:**
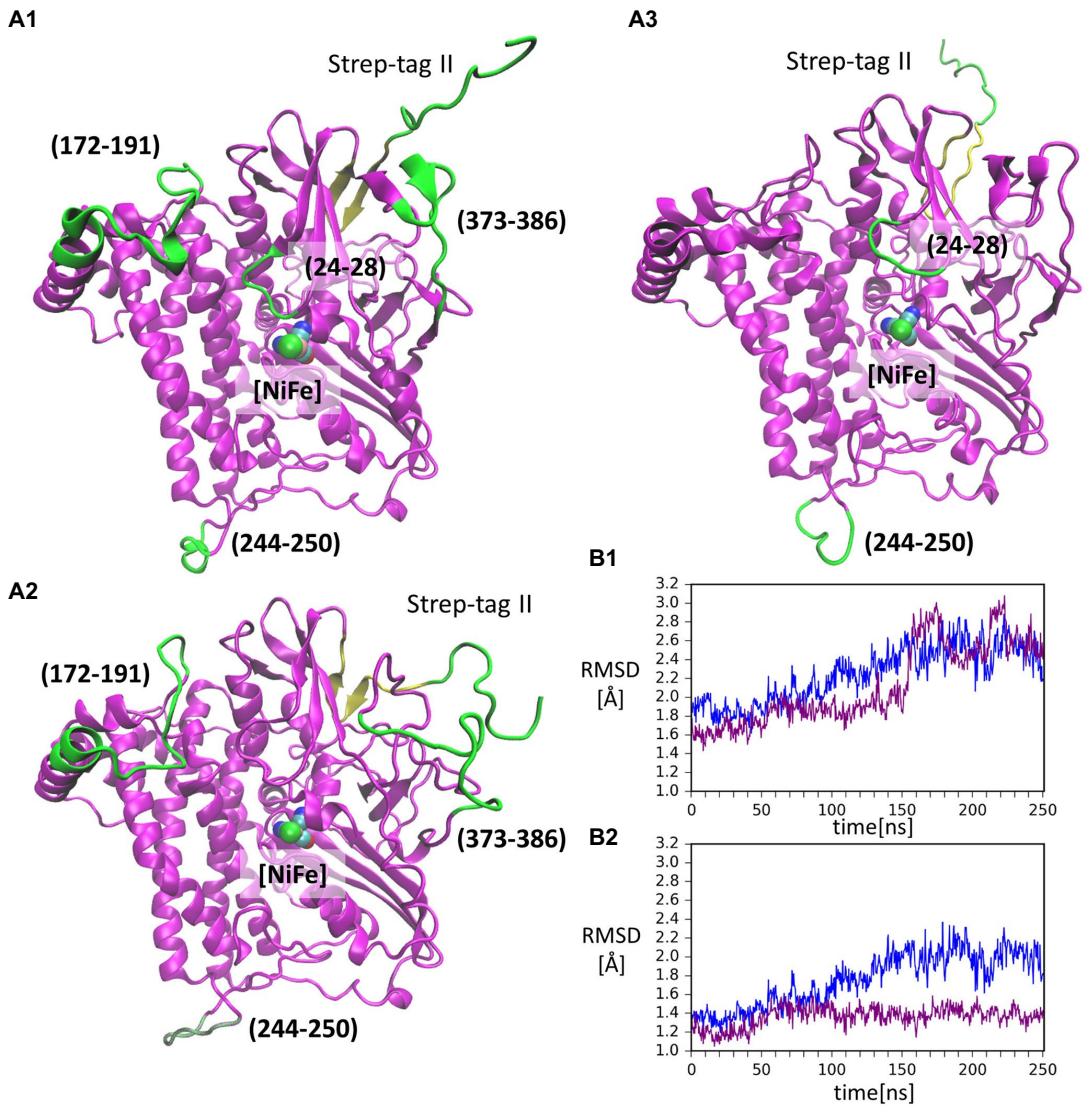
Conformations of the isolated large subunit, HoxG_m_ from GaMD simulations: **(A1)** – initial modelled structure with N-terminal Strep-tag II. Flexible loops and structure elements that were excluded from RMSD calculation in **(B2)** are shown in green; **(A2)** – representation of mobility of Strep-tag II and loops with residues 171–191, 373–386, and 244–250, shown in time frame taken from simulation 2 after 199 ns; **(A3)** – representation of mobility of Strep-tag II and loops with residues 24–28 and 244–250, shown in time frame taken from simulation 1 after 156 ns. The β-sheet adjacent to the Strep-tag II is shown in yellow in all structures. [NiFe] active site is in vdW representation in all structures. Root-mean-square deviation (RMSD) of backbone atoms relative to the crystal structure [PDB code: 3RGW (Fritsch et al., 2011)] in GaMD simulations (blue – simulation 1, purple – simulation 2): **(B1)** – Strep-tag II was excluded from the calculation; **(B2)** – Strep-tag II and all flexible loops shown in **(A1)** were excluded from the calculation.

The RMSF values of backbone Cα atoms from two GaMD simulations (shown in Supplementary Figure S1) are mostly low (below 2 Å), indicating a stable structure with some flexible loops. The highest flexibility is observed for the Strep-tag II affinity tag (Supplementary Figure S1), in line with the fact that this peptide fragment is seldom resolved in X-ray protein structures. In all simulations, no secondary structure elements could be assigned for Step-tag II, which exhibited multiple conformations relative to the HoxG structure (Supplementary Figure S2). Furthermore, the flexibility of the affinity tag may cause destabilization of the terminal β-sheet as seen in Figure 3 (residues 213, yellow). A reliable indicator of the structural stability is the root-mean-square deviation or RMSD value of the protein backbone, which was computed in all cases by excluding the mobile.

Strep-tag II (both from the alignment and the calculations). The RMSD values in cMD (Supplementary Figure S3) show a steady rise to about 2.0 Å. This was also observed for GaMD simulations, where RMSD values reached 3.0 Å (Figure 3B1). Moreover, when several flexible loops, identified based on RMSF values (residues 24–28, 171–191, 373–386, and 244–250), were excluded, the RMSD value was reduced to about 0.5 Å. The flexibility of loop regions is commonly observed in molecular dynamics simulations but it is noteworthy that residues from these loop regions form interactions with the small subunit (HoxK) in the heterodimeric MBH enzyme (Fritsch et al., 2011; Albareda et al., 2019). In contrast, these loops are solvent exposed in the HoxG_m_. Interestingly, the loop containing residues 24–28 (Figure 3A3) hosts Glu27, which plays an important proton gate role and it is located in the center of the interface of the two subunits in MBH (Tombolelli and Mroginski, 2019).

Analysis with VMD (Humphrey et al., 1996) showed that the majority of the secondary structures are preserved during both GaMD simulations, supported by a relatively low reduced RMSD, hinting at the stability of HoxG even in the absence of the small subunit. Furthermore, the relative conformational energies of time frames extracted from GaMD simulations yield average values of 345 kJ/mol and 331 kJ/mol above the 28,000 kJ/mol baseline (Supplementary Figure S4) for simulations 1 and 2, respectively, with fluctuations up to 400 kJ/mol. Despite these large fluctuations of the conformational energy, there is no indication of either protein unfolding or large conformational changes in the structures derived from GaMD simulations.

### Structural properties of the homodimer HoxG_d_

HoxG_d_ structure, predicted by SymmDock Webserver (Schneidman-Duhovny et al., 2005a,b) was subjected to cMD and GaMD simulations (see Materials and methods for details) in order to obtain a thermally equilibrated moiety for the analysis of structural features. RMSD and RMSF values (Supplementary Figures S5, S6) computed for backbone atoms indicate a high structural stability comprising a “closed” conformation and a preserved orientation of the two subunits. Computed relative conformational energies of time frames extracted from GaMD simulations yield and average value of 382 kJ/mol above the baseline value (Supplementary Figure S7), with fluctuations of around 350 kJ/mol, furthermore confirming the structural integrity of the predicted HoxG homodimer. The homodimer interface (Supplementary Figure S8) contains 52 residues (3,382 Å^2^) and 49 residues (3,366 Å^2^) belonging to HoxG-I and HoxG-II, respectively *via* 6 salt-bridges, 28 hydrogen bonds and 260 non-bonded contacts, as identified with PDBSum (Laskowski, 2009). Salt-bridges found in the thermally equilibrated HoxG_d_ after 150 ns are: Arg62(I)-Glu21(II), Arg73(I)Glu27(II), Glu21(I)-Arg62(II), Glu27(I)-Arg73(II), Arg267(I)-Asp211(II) and Glu371(I)Arg384(II). Several important residues have been observed to play a role at the interface between two HoxG subunits. Residue Glu27 that plays an important role in proton transfer in MBH is involved in the formation of a stable salt-bridge with Arg62 in both HoxG subunits in the HoxG_d_ structure (Dementin et al., 2004; Tombolelli and Mroginski, 2019). Cysteines 597 and 75, coordinating the Ni ion, are involved in interface interactions as well, albeit only observed in one subunit (HoxG-I; Supplementary Figure S8) in the HoxG_d_.

### Mechanical properties and rigidity profile of HoxG

**Figure 4:**
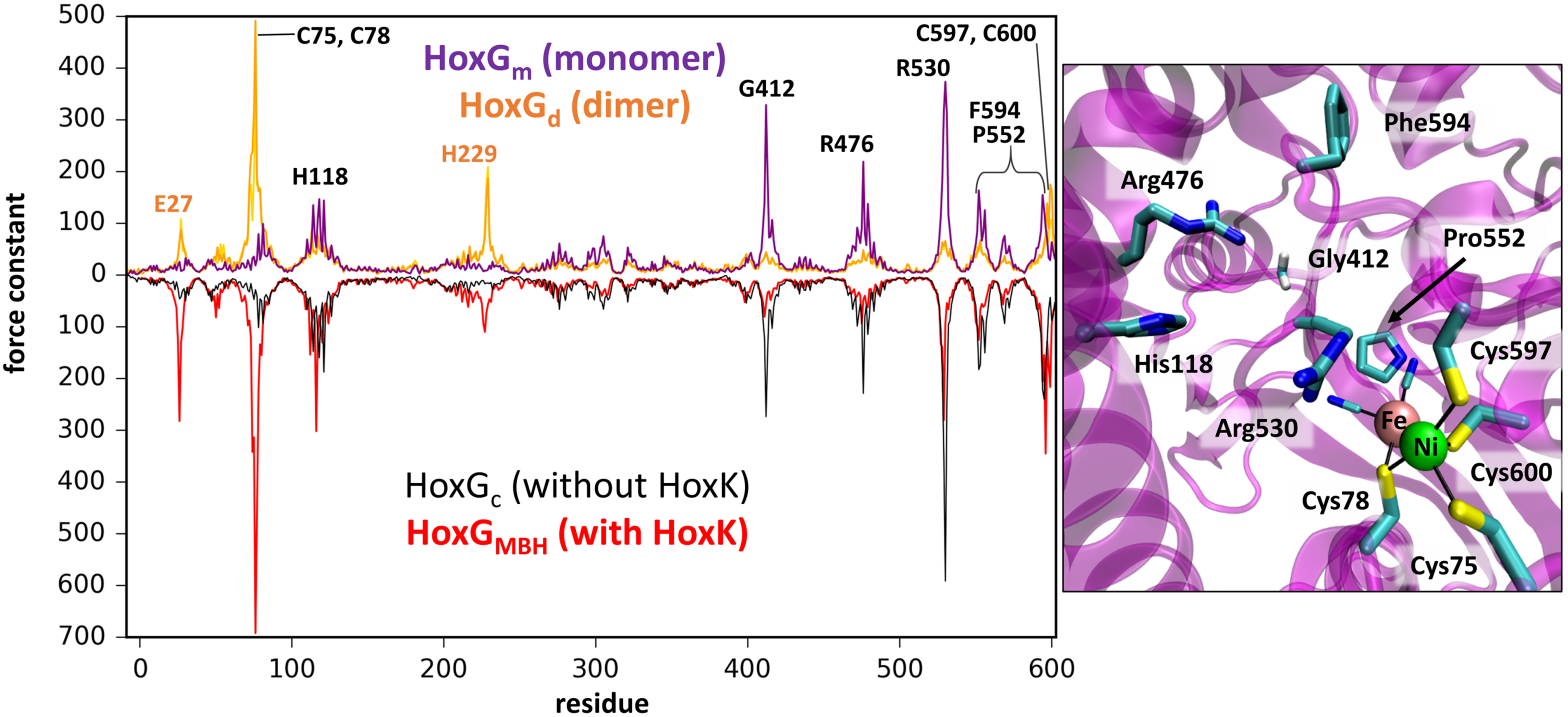
**Left**: Rigidity profiles of HoxG_m_ and HoxG_d_ (upper purple and yellow line, respectively) and HoxG_c_, HoxG_MBH_ [PDB code: 3RGW (Fritsch et al., 2011); black and red line, respectively]. Force constants (in units kcal·mol^−1^·Å^−2^) of HoxG_m_ and HoxG_d_ models were obtained using frames extracted after 200 ns GaMD simulation (simulation 2 in case of HoxG_m_). **Right**: Residues predicted with force constants of 100 kcal·mol^−1^·Å^−2^ or higher in the structures of HoxG_m_ taken from GaMD simulation 2 at 200 ns.

Computation of mechanical properties and rigidity profiles may help identify the location of the active sites in proteins (Lavery and Sacquin-Mora, 2007; Sacquin-Mora, 2016) and were utilized in this work in order to identify stable regions of the catalytic (large) subunit of MBH (HoxG) in the absence of its small counterpart HoxK. Rigidity profiles were computed for both HoxG_m_ and HoxG_d_ models based on time frames from GaMD simulations, as well as, for the entire MBH structure [PDB code: 3RGW (Fritsch et al., 2011)] and its HoxG subunit extracted from 3RGW without further processing (HoxG_c_, HoxG_MBH_; Figure 4; Supplementary Figures S9, S10).

The rigidity profile obtained for HoxG_MBH_ (Supplementary Figure S10) is qualitatively similar to those reported in an earlier study (Oteri et al., 2014a) for the soluble and membrane-bound [NiFe]-hydrogenases from *D. fructosovorans* and *A. aeolicu*s, respectively. The highest force constant peaks obtained for the MBH structure are observed for residues coordinating the active site, i.e., Cys75, Cys78, Cys597 and Cys600 (Figure 4, red line), which is a common feature in proteins with cofactors (Yang and Bahar, 2005; Sacquin-Mora and Lavery, 2006; Sacquin-Mora et al., 2007). Despite the absence of HoxK, a similar trend is observed for HoxG_c_ (Figure 4, black line) as well as in structures extracted from GaMD simulations (Figure 4, purple line), where peaks have different intensities. In the region of Cys75 and Cys78, the intensities of peaks decrease from about 700 kcal·mol^−1^·Å^−2^ to just below 100 kcal·mol^−1^·Å^−2^ and in the region of Cys597 and Cys600 the magnitude of the force constants is significantly reduced from *ca.* 300 kcal·mol^−1^·Å^−2^ to half when comparing HoxG_m_ to the HoxG_MBH_. The loss of rigidity around active site cysteines is compensated by an increase in peak intensities around residues Gly412, Arg476 and Arg530. The highest peak (at 373 kcal·mol^−1^·Å^−2^ in Figure 4, purple line) from GaMD simulations is assigned to Arg530, which is also very pronounced in the rigidity profile of HoxG_MBH_. Arg530 forms a stable salt-bridge with Asp117 in several structures of *Cn*MBH [PDB codes: 3RGW (Fritsch et al., 2011), 4IUC, 4IUB, 4IUD (Frielingsdorf et al., 2014)] and it is maintained in cMD and GaMD simulations (Supplementary Figure S11).

Generally, residues featured as peak-representatives are within 12 Å of the [NiFe] active site (Figure 4), thereby reflecting the stability of the protein core in the isolated HoxG (Figure 2). However, the [NiFe] active site in the HoxG_m_ is somewhat less rigid than that in HoxG_MBH_ with force constants of all cysteines below 100 kcal·mol^−1^·Å^−2^. Loss of rigidity around the active site may be the consequence of the solvent exposure of Ni^2+^, Cys597 and Cys75 and the increased flexibility of the adjacent loop with residues 24–28 (Figure 3A3). These sites are located at the HoxG-HoxK interface in MBH that becomes solvent exposed when isolating the HoxG unit.

Interestingly, the rigidity encompassing the [NiFe] active site, which was lost in the HoxG_m_ is partially regained in the HoxG_d_ model (Figures 2, 4). Furthermore, the rigidity of Glu27 seems to be recovered as reflected by a force constant peak of approx. 108 kcal·mol^−1^·Å^−2^, which is similar to that observed in HoxG_MBH_. His229, which interacts with the HoxK subunit in MBH, also contributes to the rigidity of the HoxG_d_ with a force constant peak of approx. 209 kcal·mol^−1^·Å^−2^. Notably, the region encompassing the Arg530 and Phe594 appears less rigid. The change of mechanical properties in this region may also have consequences on the efficiency of substrate channeling and binding to the active site.

### Electrostatic properties of HoxG_m_

The electrostatic properties of proteins are crucial for understanding their interactions with other molecules and/or surfaces, e.g., of electrodes (Oteri et al., 2014b; Heidary et al., 2015). After separation from the HoxK subunit of MBH, the electrostatic properties of the HoxG_m_ exhibit some differences compared to the HoxG_MBH_. The pK_A_ values (protonation states) initially determined with Karlsberg2^+^ (Rabenstein and Knapp, 2001; Kieseritzky and Knapp, 2008; Meyer and Knapp, 2015), remain unchanged in all MD simulations (Supplementary Table S1). Remarkable is the significant change in the pK_A_ value of Glu27 upon isolation of the HoxG subunit. This residue is located in the proximity of the [NiFe] center and is immersed in the protein matrix at the HoxG-HoxK interface. A very high pK_A_ value (> 20) is predicted, indicating a neutral charge state. Upon removal of HoxK, Glu27 becomes solvent exposed and its pK_A_ drops significantly down to *ca.* 4, suggesting the prevalence of its anionic form. Therefore, we propose that the mechanism by which protons are supplied to the active site is altered when the HoxK unit is detached from the MBH.

**Figure 5:**
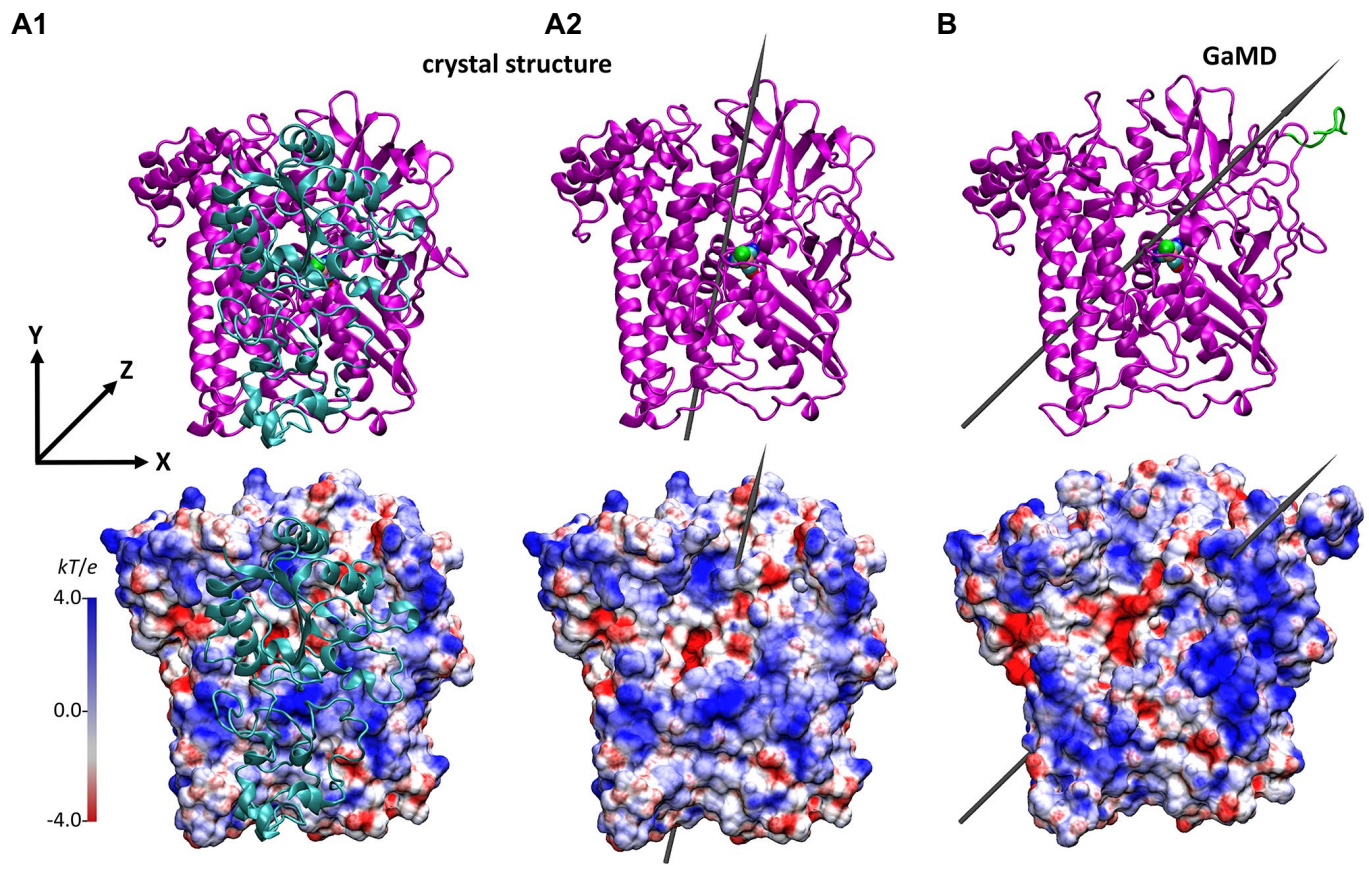
Structure and the electrostatic potential surface of the HoxG structures, showing the former dimer interface (other orientations are shown in Supplementary Figure S13). **(A)**: HoxG subunit from the MBH crystal structure 3RGW (Fritsch et al., 2011), with HoxG_MBH_ **(A1)** and without HoxG_c_ **(A2)** the small subunit HoxK. **(B)**: HoxG_m_ structure taken from simulation 2 after 100 ns. The upper panels show the secondary structure elements. The representation and color codes are taken from Figure 1. The electrostatic potential surface calculated with the APBS (Baker et al., 2001; grid resolution 0.3 Å) is qualitatively displayed (range: −4 kT/e to 4 kT/e), where red and blue indicate negatively and positively charged regions, respectively. The grey arrows indicate the direction of the dipole moment in monomers HoxG_c_ and HoxG_m_, *ca.* 644 Debye **(A2)** and *ca.* 740 Debye **(B)**, respectively. Charges and radii were used as defined in the CHARMM Force-Field (MacKerell, 1998; Best et al., 2012). Comparison to the *Cn*MBH heterodimer is shown in the SI (Supplementary Figure S12).

**Figure 6:**
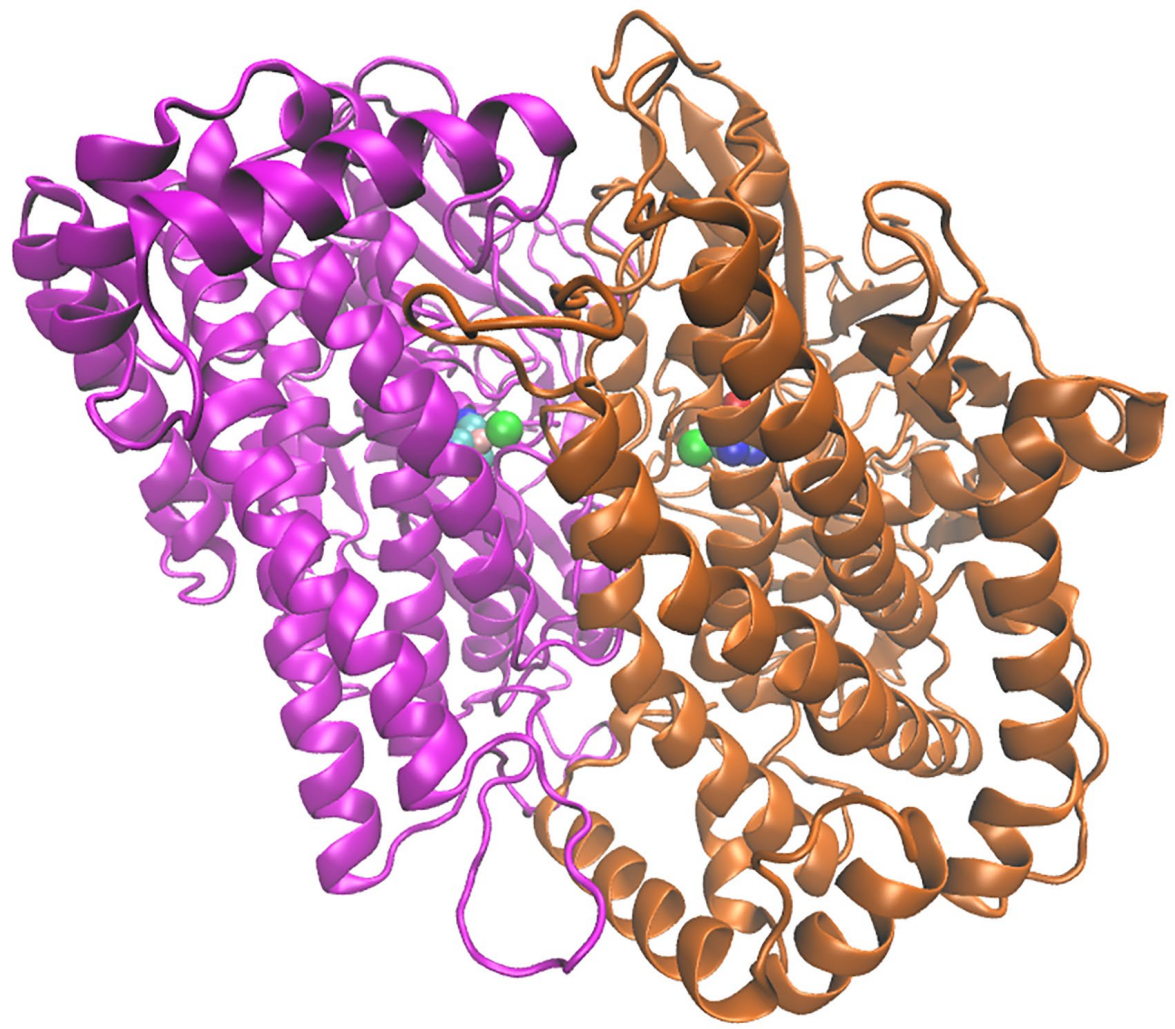
Structure of the HoxG homodimer (HoxG_d_) taken from GaMD simulation at 150 ns. 232 [NiFe]-active sites are depicted as spheres following the color code: Fe in pink, Ni in green 233 CN^−^and CO groups, cyan-blue and cyan-red, respectively.

In order to better identify changes in the electrostatic properties arising from the removal of the small HoxK subunit, we also computed the electrostatic potential surface (EPS) of the HoxG_c_ and that of HoxG_m_ using APBS (Baker et al., 2001; Figure 5). The EPS of thermally equilibrated HoxG_m_ (Figure 5B) shows a considerable number of positive charges at the solvent exposed interface (occupied by the HoxK subunit in MBH), where several Arg, Lys and His residues are present. A comparison of the computed EPS for *Cn*MBH and its HoxG subunit is shown in Supplementary Figure S12. In regions different from the former heterodimer interface we observed similar charge localizations patterns, with important exceptions in regions containing flexible loops and the Strep-tag II tag (Figure 6; Supplementary Figure S12). Here, the differences in charge distribution significantly depend on their local conformations and interactions.

The direction and magnitude of the dipole moment in HoxG_m_ were evaluated over the course of GaMD simulations using VMD (Humphrey et al., 1996), with charges and atomic radii from CHARMM ForceField (MacKerell, 1998; Best et al., 2012). The dipole moment of the HoxG_MBH_ oriented along the y axis, which was defined from Cα of Asn311 to Cα of Pro22 as reference (Figure 5A). In the course of the GaMD simulation, the total dipole moment of HoxG_m_ rotates 25° with respect to its initial position. This is shown in Figure 5B as representative conformation, taken from simulation 2 after 100 ns. Thereby, HoxG_m_ adopts an orientation similar to that in the heterodimer MBH (Supplementary Figure S12; Utesch et al., 2013; Heidary et al., 2015). Furthermore, moderate fluctuations of the magnitude and orientation of the dipole moment are predicted during the simulations (Supplementary Figure S14) like previously reported for other [NiFe]-hydrogenases (Oteri et al., 2014b).

During the GaMD simulations, the magnitude of the dipole moment of the HoxG_m_ fluctuates moderately around 800 ± 250 Debye (Supplementary Figure S14) and decreases to around 677 ± 100 Debye upon exclusion of the Strep-tag II (Supplementary Table S2). Moreover, also the orientation of the dipole moment in HoxG shows some fluctuations, as the formed angle of the dipole moment computed for HoxG_c_ in 3RGW (Fritsch et al., 2011) oscillates between 5° and 40° when the Strep Tag-II is taken into account and between 0° and 30° if the tag is absent (the related HoxG_c_ does not contain a Strep-tag II; Fritsch et al., 2011). Thus, our calculations demonstrate that the presence of a flexible tag region, carrying charged Glu, His^+^ and Lys residues, has a significant influence on the orientation and strength of the total dipole moment of HoxG protein and consequently, on the stability of the electrostatic interactions with potential reactions partners (e.g., proteins and surfaces).

### Size exclusion chromatography

**Figure 7:**
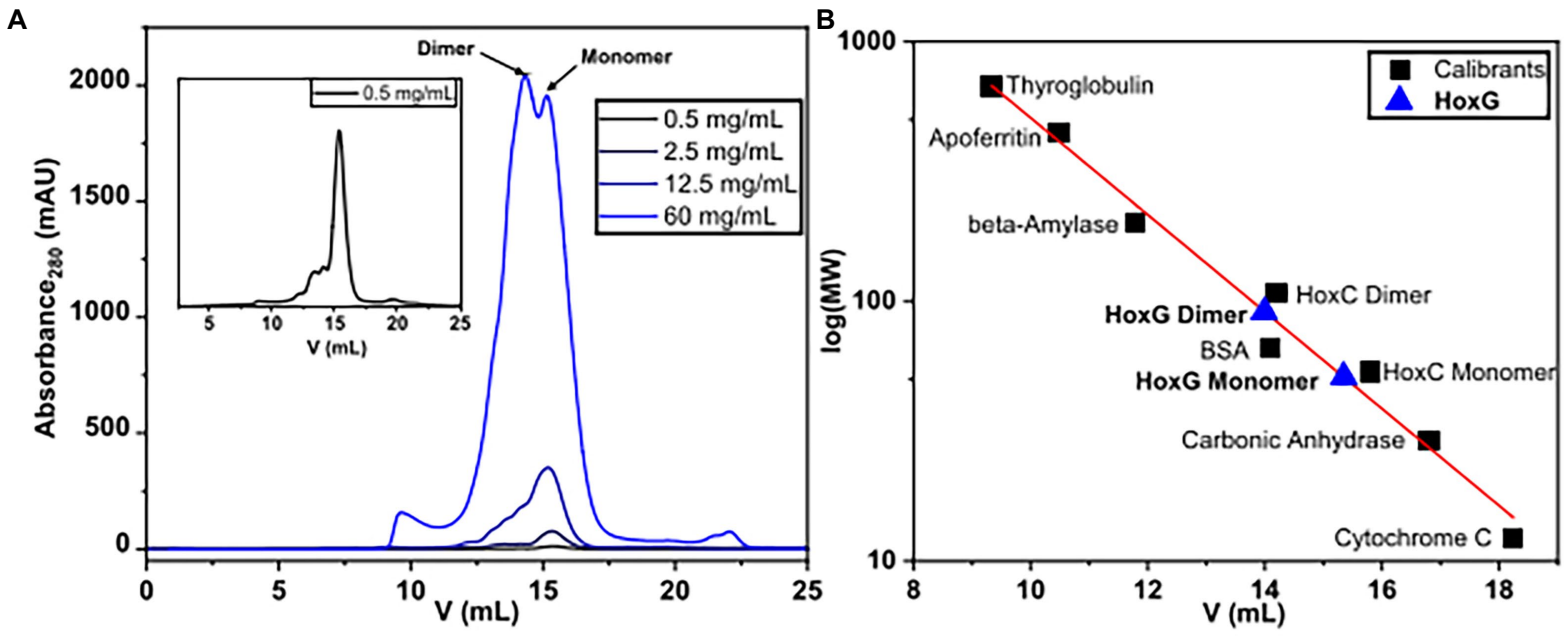
Size-exclusion chromatography measurements of HoxG in different protein concentrations **(A)** were performed in 50 mM K_i_PO4, 150 mM NaCl, pH = 7.4, T = 4°C. Monomeric and dimeric forms were calculated based on reference proteins **(B)**.

The oligomerization state of HoxG was investigated utilizing size exclusion chromatography. The protein was measured in various concentrations between 0.5 and 60 mg/ml. We observed that at concentrations below 12.5 mg/ml, the chromatographic profile of HoxG comprises mainly a monomeric form (R_V_ 15.34 ml). At higher protein concentrations, a significant amount of a dimeric form (R_V_ 14.1 ml) was detected (Figure 7).

The computational work predicted a stable HoxG_d_ homodimer at the former HoxK interface. This structural arrangement is supposed to contribute to the cofactor stability, protecting the solvent exposed NiFe(CN)_2_(CO) site from degradation. To elucidate whether the dimeric arrangement might confer stability to the active site, we used IR spectroscopy. Given that the CO and CN-diatomic ligands of the [NiFe] cofactor have specific spectroscopic signatures that vary with respect to changes in electron density at the active site, IR spectroscopy can provide detailed information on the hydrogenase cofactor monitoring redox changes, hydrogen bonding, protonation state of neighboring residues as well as stability of the [NiFe] site in the protein scaffold (Ash et al., 2017; Tai et al., 2021).

**Figure 8:**
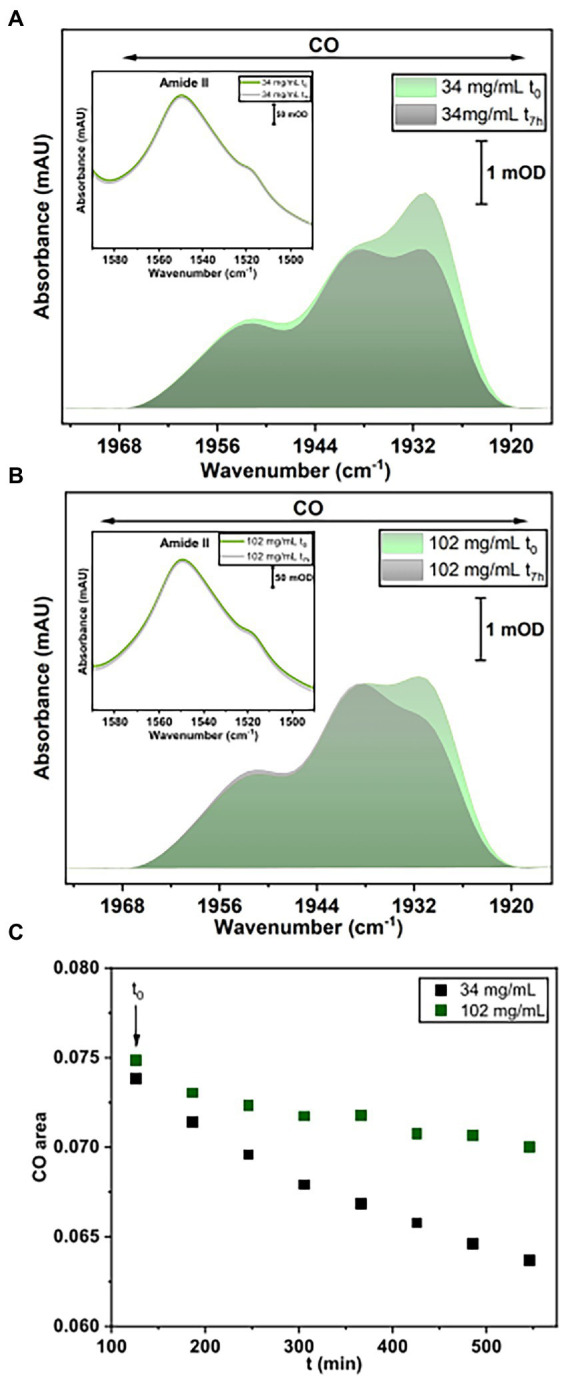
IR spectroscopy measurements. **(A)** Baseline-corrected IR spectra for HoxG sample (34 mg/ml) at t_0_ and after 7 h accumulation. **(B)** Baseline-corrected IR spectra for HoxG sample (102 mg/ml) at t_0_ and after 7 h accumulation. The spectral region in **(A,B)** displays bands related to the CO stretching vibrations of the active site. Shown spectra have been normalized to the intensity of the amide II band (see figure insets in **A,B**). **(C)** Time evolution of the integral of the [NiFe] active site CO absorption bands for the HoxG sample at 30 (black squares) and 100 mg/ml (red squares), respectively. For an adequate comparison of the time evolution of the active site signals in **A,B**, we have chosen 120 min (t_0_ = 120 min) as starting point for plotting the integral intensity of the CO absorptions.

For this purpose, IR spectra of two HoxG samples (ca 30 mg/ml and 100 mg/ml, respectively) were recorded continuously for 7 h. These two protein concentrations were chosen such that, the first sample (between 12.5 and 60 mg/ml) has a higher monomer content, while the 100 mg/ml sample has a higher dimer content. Furthermore, the comparable high protein concentrations enable a clear detection of the active site absorption bands suitable for cofactor quantitation. The spectra were first normalized to the intensity of the amide II band (at *ca.* 1,551 cm^−1^) and then the integral of the CO stretching bands region was calculated and plotted as a function of time (Figure 8A). The fully mature HoxG protein has been recently characterized (Caserta et al., 2022a) and its spectrum shows two main CO absorptions at 1,929 and 1,939 cm^−1^, and a weaker band at 1953 cm^−1^ that was assigned to an unmatured portion of the protein still containing its C-terminal peptide extension (Hartmann et al., 2018; Caserta et al., 2022a).

By comparing the first (*t*_0_) and last IR spectrum (*t*_7h_) of the two HoxG samples (Figures 8A,B) and their time dependence (Figure 8C), we observed a more pronounced loss of the active site signals in the sample with higher monomer content. Given a strong correlation between the amount of dimeric HoxG and the increased stability of the active site signals, we propose that the dimerization occurs at the interface freed by the HoxK subunit. This is in line with the computational predictions, suggesting a recovery of the active site rigidity for the HoxG protein upon (homo)-dimerization.

## Discussion

Biochemical, theoretical, and spectroscopic studies on [NiFe]-hydrogenases have focused on a small subset of these enzymes, assuming that the knowledge gained herein would apply to all [NiFe]-hydrogenases (Sickerman and Hu, 2019). Recently, we have shown that it is possible to isolate hydrogenase catalytic subunits equipped with an intact and redox-active NiFe(CN)_2_(CO) active site (HoxC and HoxG proteins from *Cn*RH and *Cn*MBH, respectively). This grants an exclusive spectral view on the hydrogenase active site without the interference from Fe-S cluster relay (Hartmann et al., 2018; Caserta et al., 2020a,b, 2021, 2022a). Albeit we suggest that these biophysical properties apply to all [NiFe]hydrogenases, the premature large subunit from *Thermococcus kodakarensis*, *Tk*HyhL (Kwon et al., 2018), was isolated exclusively in its apo-form while the catalytic subunit Pf_α_ from the cytoplasmic soluble [NiFe]-hydrogenase from *Pyrococcus furiosus* (*Pf*SHI) did not exhibit any biological and electrochemical properties (Chandrayan et al., 2015; Wang et al., 2021). Considering these recent observations and the fact that the HoxG protein has been isolated even at different stages of the biosynthetic maturation of the [NiFe] cofactor (Caserta et al., 2022a), we have undertaken an investigation of the structural and mechanical properties of this protein.

Upon removal of the small subunit HoxK from the heterodimeric MBH, the former dimer interface in the HoxG subunit becomes solvent accessible and with it, a small fraction of the catalytic site becomes exposed (Figure 2A). Alterations in the polarity of the protein environment may cause significant structural reorganization, specifically at the previous interface site. To verify this assumption, a series of classical MD simulations were performed. The changes in mechanical and electrostatic properties were evaluated using a coarse-grained Brownian dynamics approach and Poisson Boltzmann electrostatic calculations. An initial model structure for the HoxG subunit was extracted from the available crystallographic data on the heterodimeric MBH (3RGW; Fritsch et al., 2011), which was equipped with a Strep-tag II affinity tag at the N-terminus in line with the recent biochemical data (Caserta et al., 2022a). These models were thermally equilibrated with classical MD simulations and then subjected to Gaussian-Accelerated molecular dynamics GaMD (Miao et al., 2015).

Rigid docking approaches favored the formation of a HoxG homodimer (HoxG_d_), built at the interface freed by the HoxK subunit. Such protein arrangement stabilizes/protects the active site by limiting solvent accessibility. Analysis of the thermally equilibrated structures derived from GaMD simulations demonstrate that the overall HoxG_m_ structure remains stable over the course of simulations, substantiated by the relatively low RMSD (below 2.5 Å, after excluding the mobile Strep-tag II) and RMSF values (Figure 3; Supplementary Figure S1). Indeed, the core of the HoxG_m_ protein matrix with its secondary structural elements are largely preserved in our MD simulations. To shed light on the structural stability and mechanical properties, rigidity profiles of the MBH heterodimer as well as HoxG structures extracted from GaMD simulations were computed (Figure 4). The rigidity profiles of HoxG_m_ structures identified the residues Gly412, Arg476, and Arg530 in the protein core as those with the largest force constants (Figure 4). In contrast, rigidity peaks in HoxG_MBH_ are localized around active site cysteines (Cys75, Cys78, Cys597, and Cys600) consistent with those reported in earlier studies on hydrogenases (Oteri et al., 2014a). Our calculations suggest that the loss of rigidity at the [NiFe] active site of HoxG_m_ upon detachment of the HoxK subunit was most likely caused by an increased solvent accessibility around the active site. Nonetheless, biochemical and spectroscopic data revealed a stable and redox active [NiFe] active site in isolated HoxC and HoxG proteins from *C. necator* (Hartmann et al., 2018; Caserta et al., 2020a,b, 2021, 2022a). Interestingly, our computational work predicts that the rigidity around the [NiFe] site is recovered in the HoxG_d_ model, suggesting that a change in the oligomerization state of the HoxG subunit may be relevant to restore stability. These predictions were corroborated by experimental data. Indeed, size exclusion chromatography showed a concentration-dependent homodimerization of the HoxG protein and IR measurements revealed a strong correlation between the integrity of the active site and the aggregation state of the isolated HoxG (i.e., samples with higher dimer content exhibit longer remaining active absorptions as compared to the monomer counterpart). It is worth emphasizing that also other hydrogenase large subunits contain a certain amount of homodimer forms, which have not been rationalized so far (Sasaki et al., 2012; Hartmann et al., 2018; Kwon et al., 2018; Caserta et al., 2020a). Notable are in this context studies of the large subunit HyhL from *Thermococcus kodakarensis*, which was shown to form a complex at the active site interface with the Ni-inserting accessory protein HypA only upon enrichment of the large subunit monomeric form. These data suggest that the dimeric interface might be in proximity of the [NiFe] site also in *Tk*HyhL protein.

A closer look at the active site area in both HoxG_m_ and HoxG_d_ models revealed a salt-bridge between Arg530 and Asp117 that is conserved also in multiple MBH crystal structures (Fritsch et al., 2011; Frielingsdorf et al., 2014). The Arg530 has been shown to play a relevant functional and structural role in [NiFe]hydrogenases. Indeed, this Arg residue located above the free coordination site of the [NiFe]center (Supplementary Figure S11) has been proposed to act as general base triggering H_2_ activation according to a frustrated Lewis pair mechanism (Evans et al., 2016). The Arg530 in HoxG_m_ is associated with the highest peak in the rigidity profile, indicating that this residue plays a significant role in stabilizing the surrounding protein matrix.

In previous works done on the MBH heterodimer (Smith et al., 2012; Utesch et al., 2013; Heidary et al., 2015; Kalms et al., 2018), no large conformational changes of HoxG (or HoxK) were observed and our new simulations reveal preservation of the mechanical properties in HoxG_m_. This statement is supported by the steady temporal evolution of the conformational energies of HoxG_m_ structures obtained over the course of the GaMD simulations (Supplementary Figure S4). These data do not show drastic conformational changes in HoxG_m_, which could eventually lead to protein unfolding. Moreover, the former heterodimer interface of HoxG_m_ is largely hydrophilic as can be seen in the map of electrostatic potential (Figure 5). Herein, the positive charges from Lys, His^+^ and Arg residues support the overall stability of the protein in aqueous solution.

In addition to the highly dynamic Strep-tag II (Supplementary Figure S1), several flexible loop regions (Figure 6A1) were identified on the edges of the new solvent-exposed interface. In the HoxG_MBH_, residues from these flexible regions form non-covalent interactions with the small subunit HoxK (Albareda et al., 2019). Herein, we propose that the flexible regions could be relevant for interactions of the HoxG_m_ with other molecular species such as maturase proteins involved in the biosynthesis of the [NiFe] cofactor (Lacasse and Zamble, 2016; Kwon et al., 2018) and may be used to facilitate the protein immobilization on electrode surfaces (Utesch et al., 2013). In the latter case, detailed information on the electrostatic potential surface can guide the optimization of protein immobilization strategies assuring an efficient electrochemical control. In this regard, early work on MBH heterodimer using (spectro)electrochemical techniques showed that immobilization of the enzyme can be controlled by changing the protonation of the self-assembled monolayer (SAM) of functionalized n-alkanethiols attached to the gold surface (Utesch et al., 2013).

In summary we have clearly shown that a Strep-tag II sequence, often included to enhance protein purity and homogeneity (Johar and Talbert, 2017), has a substantial effect on the electrostatic behavior of a macromolecule (Oteri et al., 2014b). In the case of the HoxG_m_, this highly flexible positively charged sequence of amino acids is responsible for destabilizing the dipole moment and reducing its strength. However, the affinity tag does not control the dipole moment direction as much as the rest of the protein (Supplementary Figure S14). Generally, the direction of the dipole moment in HoxG_m_ (avg. value *ca.* 833 Debye; Supplementary Table S2) as depicted in Supplementary Figure S12, is steady throughout the MD simulations.

Finally, our combined computational/experimental data revealed that the artificial isolation of the large subunit of MBH does not result in protein unfolding and the key mechanical properties are preserved. Various oligomers could be observed in HoxG_m_ depending on the protein concentration, and we propose that homodimers are formed *via* the former HoxG-HoxK interface of the MBH. This arrangement confers mechanical stability to the active site and we hypothesize that the active site rigidity may be regained also through a specific and oriented immobilization of HoxG_m_ on a functionalized surface, as previously observed in the case of similar enzymes (Oteri et al., 2014b). More importantly, we used thermally equilibrated structures from GaMD simulations to determine formerly unknown properties of HoxG_m_, such as surface charge distribution and dipole moment strength and orientation. This information is essential for understanding the details of the hydrogenase maturation (Lacasse and Zamble, 2016; Hartmann et al., 2018; Caserta et al., 2020a, 2022a), achieving electrostatic control of these enzymes and more importantly boosting their applications.

## Data availability statement

The original contributions presented in the study are included in the article/Supplementary material, further inquiries can be directed to the corresponding author.

## Author contributions

JD and SS-M: calculations. CK-R, SK, and GC: experiments. JD, SS-M, SK, GC, IZ, and MM: analysis, writing—original draft preparation, and writing—review and editing. IZ, OL, and MM: funding acquisition. All authors contributed to the article and approved the submitted version.

## Funding

This work was funded by the Deutsche Forschungsgemeinschaft (DFG, German Research Foundation) under Germany’s Excellence Strategy – EXC 2008–390540038 (UniSysCat), further financial support was granted by the “Initiative d’Excellence” program from the French State (Grant “DYNAMO,” ANR-11-LABX-0011-01).

## Conflict of interest

The authors declare that the research was conducted in the absence of any commercial or financial relationships that could be construed as a potential conflict of interest.

## Publisher’s note

All claims expressed in this article are solely those of the authors and do not necessarily represent those of their affiliated organizations, or those of the publisher, the editors and the reviewers. Any product that may be evaluated in this article, or claim that may be made by its manufacturer, is not guaranteed or endorsed by the publisher.
