# Supplementary material for: Conformational and mechanical stability of the isolated large subunit of membrane-bound [NiFe]-hydrogenase from Cupriavidus necator
Source: Front Microbiol. 2023 Jan 17;13:1073315. doi: 10.3389/fmicb.2022.1073315 (PMC9886862; doi:10.3389/fmicb.2022.1073315)
Supplement: Supplementary file 1 [file Data_Sheet_1.pdf]

Supplementary Information

Conformational and Mechanical Stability of the Isolated Large Subunit of Membrane-Bound [NiFe]-Hydrogenase from *Cupriavidus necator*

Jovan Dragelj[a], Chara Karafoulidi-Retsou[a], Sagie Katz[a], Oliver Lenz[a], Ingo Zebger[a], Giorgio Caserta[a], Sophie Sacquin-Mora[a,b,c], Maria Andrea Mroginski[a]

[a] Institut für Chemie, PC 14 Technische Universität Berlin, Straße des 17. Juni 135, 10623 Berlin, Germany.

[b] CNRS, Université de Paris Cité, UPR 9080, Laboratoire de Biochimie Théorique, 13 rue Pierre et Marie Curie, 75005 Paris, France.

[c] Institut de Biologie Physico-Chimique-Fondation Edmond de Rotschild, PSL Research University, Paris, France.

Table of contents

Table S1. Average pK_A values of titratable residues in $HoxG_m$ and $HoxG_{MBH}$ 3

Table S2. Average $HoxG_m$ dipole moment intensities 7

Figure S1. Root-mean-square fluctuation (RMSF) of Cα-backbone atoms of $HoxG_m$ 8

Figure S2. Flexibility of the N-terminal Strep-tag II 9

Figure S3. Root-mean-square deviation (RMSD) of backbone atoms in cMD 9

Figure S4. Conformational energy over time of $HoxG_m$ in GaMD 10

Figure S5. Root-mean-square fluctuation (RMSF) of Cα-backbone atoms of $HoxG_d$ 10

Figure S6. Root-mean-square deviation (RMSD) of backbone atoms of $HoxG_d$ 11

Figure S7. Conformational energy over time of $HoxG_d$ in GaMD 11

Figure S8. $HoxG_d$ structure and interface residues 12

Figure S9. Rigidity profiles of the $HoxG_m$, $HoxG_{MBH}$ and $HoxG_c$ 13

Figure S10. Rigidity profile of the MBH heterodimer… 13

Figure S11. Arg530-Asp117 salt-bridge conformation 14

Figure S12. Electrostatic potential surface and dipole moment of MBH and the $HoxG_m$………………15

Figure S13. Electrostatic potential surface of $HoxG_m$ in different orientations 16

Figure S14. Dipole moment intensity and direction (angle) over time... 17

References.. 18

In the following, models of the large subunit HoxG will be labelled as follows: $HoxG_m$: thermodynamically equilibrated monomeric form, $HoxG_d$: thermodynamically equilibrated homodimeric form, $HoxG_c$: isolated HoxG in crystallographic arrangement; $HoxG_{MBH}$: HoxG complexed with HoxK (small subunit) in crystallographic arrangement.

Table S1. Average pK_A values and standard deviations (pK_A-SD) of titratable residues in the $HoxG_m$ computed from Gaussian accelerated (GaMD) [1] simulations. These values are compared to those from MBH heterodimer ($HoxG_{MBH}$). Residues labelled with prefix "tag" belong to the N-terminal Strep-tag II sequence. Histidine tautomers are indicated in brackets (δ or ε). Karlsberg2$^+$ (KB2$^+$) [2]–[4] software was used.

		**GaMD simulation 1**		**GaMD simulation 2**		**$HoxG_{MBH}$[5]**
**Residue**	**Prot. State**	**pK_A**	**pK_A-SD**	**pK_A**	**pK_A-SD**	**pK_A**
tag-His6	charged	6.93	1.05	8.92	2.83	N/A
tag-Glu10	charged	4.29	0.81	2.87	2.35	N/A
Lys1	charged	11.13	1.49	14.35	2.70	N/A
Tyr4	neutral	14.25	2.64	11.87	2.10	17.09
Asp12	charged	-3.56	4.29	-4.34	2.18	-3.61
Asp13	charged	3.78	1.92	1.68	2.42	3.64
Arg14	charged	15.37	1.90	14.82	0.74	14.06
Arg16	charged	13.15	1.48	13.03	1.37	13.98
Arg17	charged	17.84	2.11	17.50	1.11	>20.00
Asp21	charged	0.33	2.75	-4.70	5.05	<-10.00
Arg25	charged	15.18	2.75	>20.00	0.00	>20.00
Glu27	charged	4.02	0.46	4.32	0.45	>20.00
His29	neutral (ε)	4.76	1.39	4.66	1.10	0.15
Arg31	charged	19.47	0.90	19.43	0.88	>20.00
Cys32	neutral	>20.00	0.00	>20.00	0.00	>20.00
Glu33	charged	-6.16	3.04	-4.58	1.91	<-10.00
Asp37	charged	3.15	1.45	0.55	3.37	2.72
Arg43	charged	14.63	1.94	13.82	0.95	12.59
Arg53	charged	13.91	2.24	12.50	0.67	>20.00
Glu56	charged	<-10.00	0.00	<-10.00	0.00	<-10.00
Lys60	charged	10.29	0.85	10.94	1.45	7.85
Arg62	charged	12.31	0.41	12.41	0.45	19.74
Asp63	charged	-4.84	2.78	-4.79	4.01	-7.59
Arg65	charged	15.17	1.40	15.73	2.04	>20.00
Asp66	charged	3.97	0.36	3.98	0.80	<-10.00
Glu72	neutral	9.77	4.37	7.34	2.09	>20.00
Arg73	charged	12.93	0.71	12.75	0.85	>20.00
Cys81	neutral	>20.00	0.00	>20.00	0.00	>20.00

His82	neutral(ε)	-8.82	2.72	-8.24	3.43	-0.92
Arg88	charged	19.63	0.98	19.98	0.17	>20.00
Glu91	charged	-9.98	0.21	<-10.00	0.05	<-10.00
Asp95	charged	2.78	2.35	2.59	2.51	2.47
Arg97	charged	14.28	0.89	14.30	0.90	14.13
Lys100	charged	11.68	1.12	12.60	1.51	10.37
His103	neutral(ε)	1.29	2.02	1.82	2.28	2.00
Arg106	charged	>20.00	0.00	>20.00	0.00	>20.00
Glu107	charged	-1.67	4.28	-0.64	3.08	-4.45
Lys111	charged	>20.00	0.00	>20.00	0.00	>20.00
His116	neutral(ε)	2.87	1.94	-0.05	3.01	<-10.00
Asp117	charged	-9.34	2.37	-8.90	2.66	<-10.00
His118	charged	12.15	4.14	11.28	5.24	14.75
His121	neutral(ε)	-9.37	2.34	-7.64	2.87	<-10.00
Tyr123	neutral	>20.00	0.00	>20.00	0.00	>20.00
His124	neutral(ε)	0.41	3.04	3.91	1.31	<-10.00
His126	neutral(ε)	-7.71	3.03	-9.98	0.12	<-10.00
Asp129	charged	-2.53	5.29	<-10.00	0.00	<-10.00
Asp132	charged	0.12	1.53	0.05	1.05	-1.09
Lys138	charged	10.58	0.23	10.49	0.25	10.75
Asp140	charged	-1.99	3.00	-1.55	2.79	-0.24
Lys142	charged	12.79	1.86	12.92	1.89	10.77
Arg143	charged	13.91	1.09	13.68	1.26	14.11
Glu146	charged	2.79	1.86	3.54	0.99	5.09
His155	neutral(ε)	1.14	1.95	0.24	1.34	2.07
Tyr162	neutral	15.17	2.74	18.36	2.54	>20.00
Arg164	charged	12.44	0.95	12.44	0.70	11.53
Asp165	charged	2.98	1.79	3.40	1.60	4.31
Arg169	charged	13.45	1.20	13.07	1.09	16.04
Lys171	charged	11.84	1.63	11.84	1.50	10.54
Arg172	charged	12.38	0.81	12.72	1.12	12.99
Glu175	charged	2.58	1.70	2.03	2.23	4.73
Tyr186	neutral	12.13	2.63	15.95	4.30	>20.00
Lys190	charged	10.76	0.88	10.54	0.23	10.71
Tyr192	neutral	14.98	2.64	>20.00	0.02	>20.00
Glu197	charged	3.61	0.77	3.08	0.81	3.44
His205	charged	17.34	2.06	18.12	1.54	>20.00
Tyr206	neutral	15.87	2.67	15.42	1.01	>20.00
Glu208	charged	5.34	1.33	4.60	1.64	-3.97
Asp211	charged	3.85	1.15	4.14	1.16	-4.26
Lys214	charged	12.37	2.51	12.01	2.29	16.96
Glu215	charged	-1.58	3.13	-0.33	4.73	-5.09
Lys218	charged	13.47	2.04	13.26	2.11	>20.00

His220	neutral(ε)	-6.59	3.17	-6.64	2.81	<-10.00
Lys226	charged	10.75	1.00	11.92	1.79	>20.00
His229	neutral(ε)	2.61	3.13	0.65	2.88	<-10.00
Tyr232	neutral	19.76	0.79	18.66	1.45	>20.00
Cys239	neutral	18.93	1.27	18.13	1.72	>20.00
Asp244	charged	3.96	0.63	3.86	0.72	3.81
Glu256	charged	3.53	1.71	3.27	2.60	-3.21
Arg257	charged	12.18	1.33	12.44	1.14	15.84
Lys262	charged	15.69	0.97	15.54	1.17	13.29
Arg264	charged	15.13	1.80	16.17	1.41	15.58
Asp266	charged	-2.86	2.14	-2.99	2.16	-1.33
Glu267	charged	3.29	2.33	1.25	3.16	0.51
Glu270	charged	1.11	3.22	3.72	2.62	4.22
Lys273	charged	15.39	2.84	12.83	2.34	13.18
Tyr276	neutral	>20.00	0.00	>20.00	0.00	>20.00
Asp279	charged	-9.74	0.70	-9.90	0.32	-3.85
Tyr287	neutral	18.05	2.57	15.48	2.04	19.40
Lys288	charged	13.76	4.16	15.32	3.55	9.36
Tyr294	neutral	11.15	0.90	10.90	0.41	10.95
Asp305	charged	-9.04	1.48	-9.58	0.75	<-10.00
Tyr306	neutral	19.45	0.71	19.65	0.58	>20.00
Glu308	charged	-7.37	3.98	-9.35	2.04	<-10.00
Tyr309	neutral	>20.00	0.00	>20.00	0.00	>20.00
Tyr314	neutral	11.27	1.63	11.46	1.42	11.30
Lys316	charged	11.77	1.76	11.25	0.92	19.83
Asp319	charged	3.08	2.32	3.46	1.44	3.48
Asp332	charged	3.21	1.30	3.35	1.11	2.56
Glu333	charged	4.74	0.32	4.83	0.33	5.35
Asp338	charged	-6.56	3.07	-6.02	3.30	0.85
Arg340	charged	15.16	0.78	15.14	0.70	13.08
Asp341	charged	1.60	0.76	1.52	0.80	0.83
Glu347	charged	-9.89	0.74	-9.83	1.01	<-10.00
His351	charged	7.74	1.01	8.13	1.70	7.18
Tyr354	neutral	17.53	2.27	15.74	2.88	>20.00
Lys355	charged	12.26	1.51	12.82	1.60	11.91
Tyr356	neutral	19.34	1.23	19.48	1.27	>20.00
Asp358	charged	2.11	0.69	1.58	1.78	3.43
Glu359	charged	-0.95	2.57	-1.33	2.73	-2.06
His364	neutral(ε)	-4.93	1.62	-4.96	1.77	-6.14
Asp367	charged	3.71	0.43	3.70	0.64	2.57
Glu371	charged	2.89	1.14	2.79	1.66	3.50
Tyr374	neutral	14.59	1.94	12.60	2.60	>20.00
Lys381	charged	11.10	1.04	10.77	0.96	10.32

Arg384	charged	12.17	0.84	12.30	1.22	14.37
Arg386	charged	13.00	1.69	14.15	1.50	12.78
Glu388	charged	3.79	1.29	2.88	2.21	3.80
Asp391	charged	-0.81	1.44	-0.66	3.41	-0.12
Glu392	charged	1.58	4.81	3.77	2.44	-5.92
Lys395	charged	19.16	1.87	19.03	2.19	>20.00
Tyr396	neutral	19.99	0.04	19.97	0.14	>20.00
Lys400	charged	19.46	1.62	19.36	2.06	12.51
Arg403	charged	>20.00	0.00	>20.00	0.00	>20.00
Arg405	charged	12.08	0.34	12.08	0.35	12.13
His407	neutral(ε)	4.71	0.81	4.69	0.80	5.01
Glu410	charged	<-10.00	0.00	<-10.00	0.00	<-10.00
Arg416	charged	>20.00	0.00	>20.00	0.00	>20.00
Tyr417	neutral	>20.00	0.00	>20.00	0.00	>20.00
Tyr421	neutral	11.71	1.79	11.88	2.05	11.49
His423	neutral(ε)	2.94	2.19	2.52	2.01	5.06
Arg425	charged	13.98	1.48	14.20	1.61	14.14
Lys429	charged	12.60	1.68	12.47	1.63	10.63
Tyr430	neutral	12.07	0.81	12.17	0.89	12.38
Glu432	charged	2.71	2.24	2.54	2.26	-2.32
Arg433	charged	17.26	1.86	16.74	2.29	19.08
Lys435	charged	15.31	1.87	15.25	2.10	16.16
Glu436	charged	3.47	1.18	2.98	1.48	3.40
Glu439	charged	1.37	1.22	1.56	1.23	5.54
Tyr440	neutral	10.95	0.74	11.19	0.93	11.20
Lys451	charged	12.91	1.56	13.46	1.37	11.05
Glu457	charged	1.57	2.21	0.95	2.15	3.85
Tyr460	neutral	11.75	1.52	10.73	0.94	8.50
Lys463	charged	10.74	0.76	10.78	0.79	9.95
Arg472	charged	>20.00	0.00	>20.00	0.00	>20.00
Arg476	charged	19.87	0.65	19.88	0.54	>20.00
Glu479	charged	<-10.00	0.00	<-10.00	0.00	<-10.00
Tyr482	neutral	19.97	0.24	19.85	0.70	>20.00
Cys483	neutral	>20.00	0.00	>20.00	0.00	>20.00
Glu485	charged	-3.27	4.19	-0.63	4.62	-7.47
His488	neutral(δ)	1.71	2.83	1.60	2.75	3.00
Asp490	charged	-5.21	2.52	-5.18	2.87	-5.13
His492	neutral(ε)	5.81	0.70	5.72	0.81	5.14
Asp493	charged	4.97	0.95	5.11	1.21	5.24
Arg499	charged	12.45	0.36	12.50	0.39	12.31
Asp502	charged	3.56	0.78	2.64	1.55	2.62
Asp509	charged	3.71	0.86	3.72	0.61	4.84
Lys510	charged	11.13	0.84	11.31	0.99	13.09

Asp512	charged	3.82	0.72	3.52	0.56	2.69
Lys521	charged	10.31	0.36	10.28	0.46	9.79
Arg530	charged	>20.00	0.00	>20.00	0.00	>20.00
His535	neutral(ε)	-9.70	1.40	-9.92	0.67	<-10.00
Arg538	charged	14.29	1.42	14.28	1.29	14.86
Lys540	charged	14.06	1.32	13.63	1.36	11.20
Asp541	charged	1.21	1.99	1.72	1.95	4.36
Arg543	charged	12.88	0.67	12.97	0.80	13.77
Glu545	charged	0.24	3.55	1.07	2.15	3.91
Tyr547	neutral	19.86	0.61	19.69	0.88	>20.00
Cys549	neutral	>20.00	0.00	>20.00	0.00	>20.00
Arg560	charged	18.50	1.64	>20.00	0.00	>20.00
Asp561	charged	-9.96	0.38	-9.88	0.43	<-10.00
Tyr562	neutral	10.71	0.57	11.25	1.80	11.41
Lys563	charged	10.56	0.59	10.71	0.60	9.80
Glu570	charged	0.07	2.97	-8.60	1.75	<-10.00
Glu582	charged	3.41	1.96	3.66	1.63	2.86
Glu586	charged	-3.02	3.54	1.15	1.95	0.20
Arg589	charged	18.65	1.81	19.63	1.21	>20.00
His592	charged	10.48	5.35	13.59	2.12	>20.00
Asp595	charged	-2.41	3.76	-6.70	3.93	<-10.00
His603	neutral(δ)	<-10.00	0.00	<-10.00	0.00	<-10.00

Table S2. Average dipole moment intensities obtained from Gaussian accelerated (GaMD) [1] simulations (in D, Debye).

	**simulation 1**	**simulation 2**
**HoxG$_m$ with Strep-tag II**	815.0	849.4
**HoxG$_m$ without Strep-tag II**	677.8	727.1

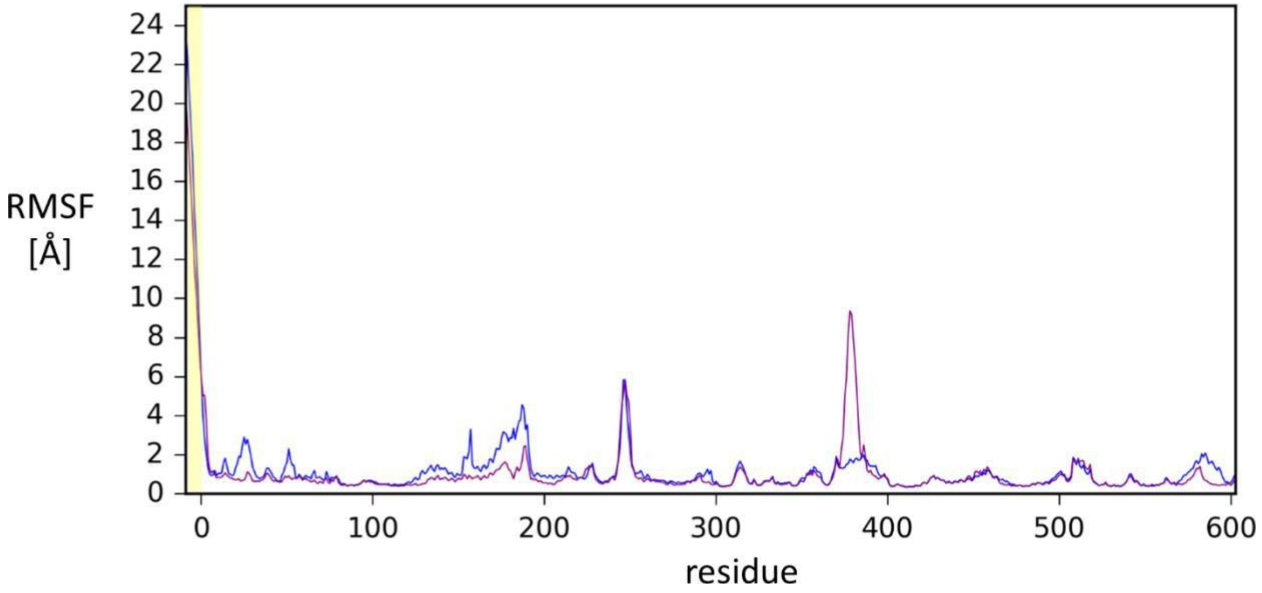

Figure S1. Root-mean-square fluctuation (RMSF) of Cα-backbone atoms of $HoxG_m$ residues during 250 ns GaMD simulations (simulation **1** in blue and simulation **2 in** purple) after the alignment to the $HoxG_{MBH}$ in the crystal structure (3RGW) [5]. The strep-tag II sequence was excluded from the alignment. Strep-tag II residues are found in the region shaded in yellow, where number 0 corresponds to the Glu residue of the Strep-tag II sequence.

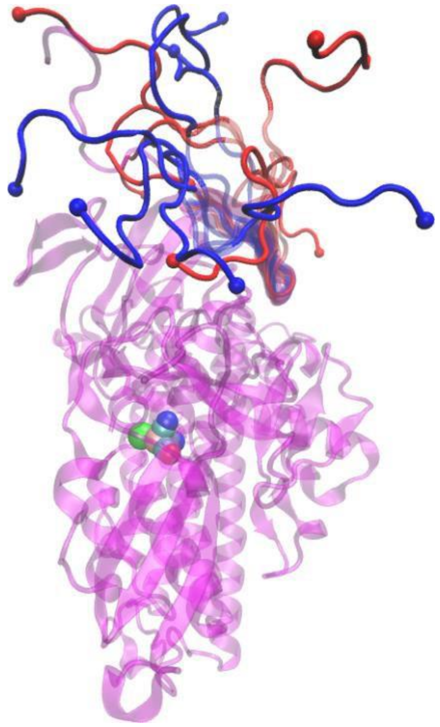

Figure S2. Flexibility of the N-terminal Strep-tag II attached to the large subunit $HoxG_m$ taken from GaMD simulations (simulation **1** inred and simulation **2** in blue) at time intervals of 50 ns. The structures were aligned to the $HoxG_{MBH}$ in the crystal structure (3RGW) [5] (shown in magenta, transparent). [NiFe] active site is shown in vdW representation with the color scheme from Figure 1. Ball and stick representation was used to mark the Cα-backbone atoms of the first residue of Strep-tag II sequence.

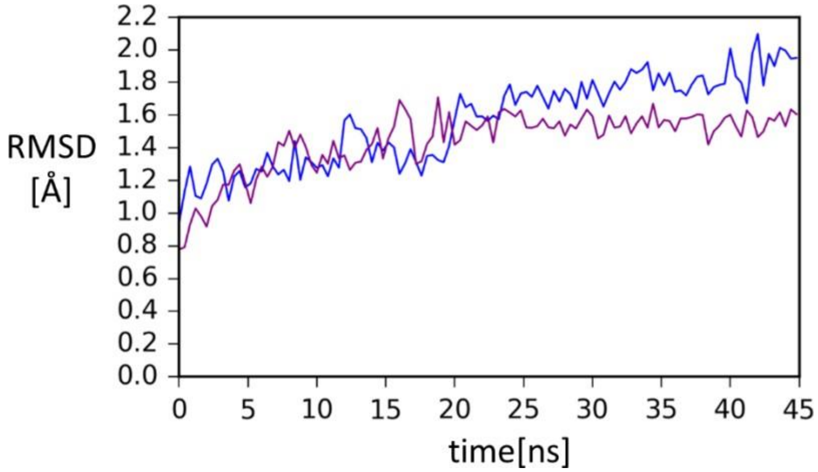

Figure S3. Root-mean-square deviation (RMSD) of backbone atoms in $HoxG_m$ in cMD (blue - simulation **1**, purple – simulation **2**) relative to the $HoxG_{MBH}$ in the crystal structure (3RGW) [5]. Strep-tag II was excluded from the alignment and calculations

.

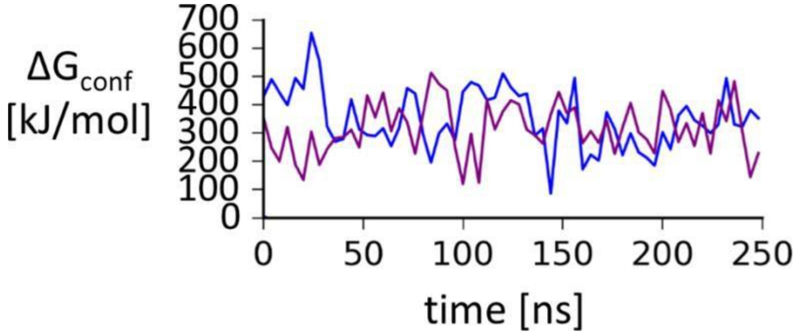

Figure S4. Conformational energy (ΔG_{conf}) of $HoxG_m$ computed with APBS [6] based on time frames of 250 ns GaMD simulations (blue – simulation **1**, purple – simulation **2**). The values are relative to the baseline value of 28300 kJ/mol.

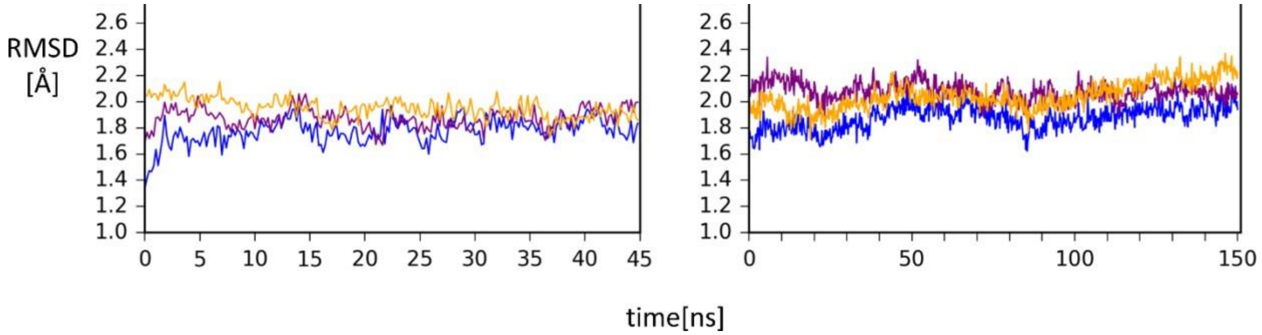

Figure S5. Root-mean-square deviation (RMSD) of backbone atoms in $HoxG_d$ from cMD (Left) and GaMD (right). In blue are depicted RMSD values over the course of simulations relative to the initial dimer model generated by SymmDock Webserver [7], [8]. The RMSD values for two subunits (orange and purple) were calculated relative to the $HoxG_{MBH}$ in the crystal structure [5].

Strep-tag II was excluded from the alignment and calculations.

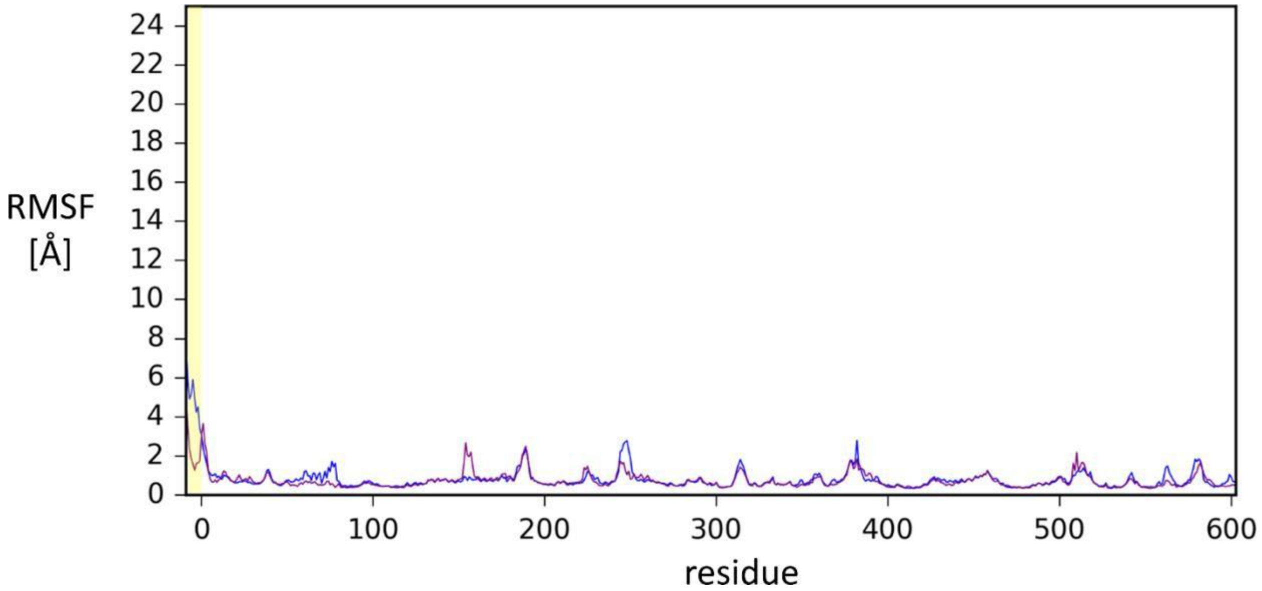

Figure S6. Root-mean-square fluctuation (RMSF) of Cα-backbone atoms of the residues in the two subunits (blue and purple) of the $HoxG_d$ during 150 ns GaMD simulations after the alignment to the initial dimer model generated by SymmDock Webserver [7], [8]. Strep-tag II was excluded from the alignment. Strep-tag II residues are found in the region shaded in yellow, where number 0 corresponds to the Glu residue of the Strep-tag II sequence.

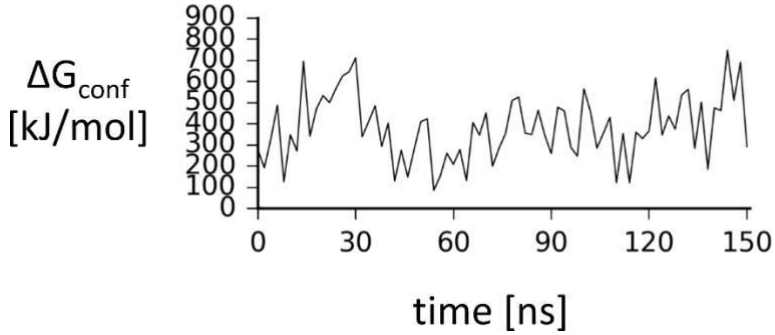

Figure S7. Conformational energy (ΔG_{conf}) of the $HoxG_d$ computed with APBS [6] based on time frames of 150 ns GaMD simulations. The values are relative to the baseline value of 56200 kJ/mol.

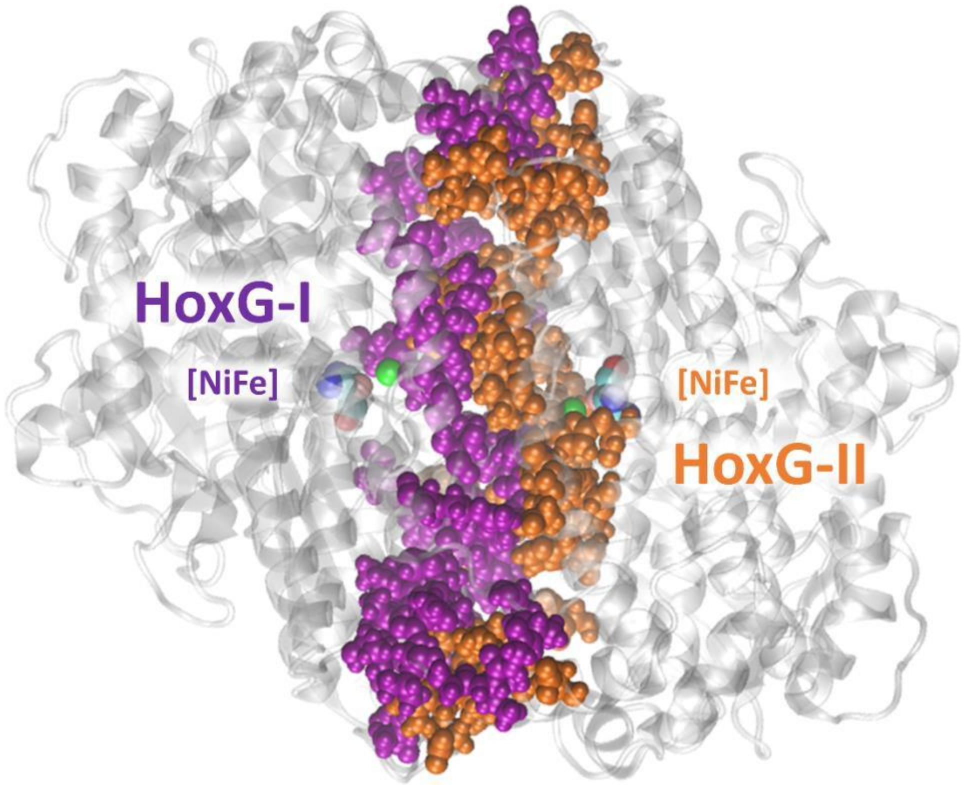

Figure S8. HoxG$_d$ structure after 150 ns of GaMD simulation. Protein backbone is transparent and interface residues identified by PDBSum [9] are depicted in magenta (HoxG I subunit) and orange (HoxG II subunit) colors. Residues identified at the HoxG$_d$ dimer interface: A) **HoxG-I subunit (magenta)**: Arg65, Leu233, Gly225, Pro238, Gly247, Ile246, Gly245, Ala248, Ala506, Ser250, Thr505, Asn242, Cys239, Ala240, Arg257, Thr503, Asn231, Cys597, Leu243, Val237, Lys226, Asn227, Phe70, Arg62, Val508, Ala69, Thr221, Lys60, Arg73, Lys214, Arg53, Cys75, Glu271, Gly28, Glu27, Ile78, Gln213, His124, Val57, Phe173, Gln178, Arg169, Arg384, His29, Arg386, Val217, Pro22, Asp21, Asn184, Ser176, Glu175, Gly177. **B) HoxG-II subunit (orange)**: Met183, Asn184, His116, Asp165, Arg169, Phe173, Ser176, Gln178, Asp211, Val120, His124, Thr602, Gln213, Leu598, Ile26, Asp21, Val217, Arg172, Glu27, Arg53, Val77, His29, Ala599, Gly76, Met51, Thr221, Arg73, Arg384, Glu175, Phe70, Lys226, Pro238, Pro372, Thr385, Thr50, Val237, Ala249, Ile58, Ser250, Asn242, Arg62, Cys239, The505, Val508, Arg65, Asn227, Trp511, Ala248, Ala506.

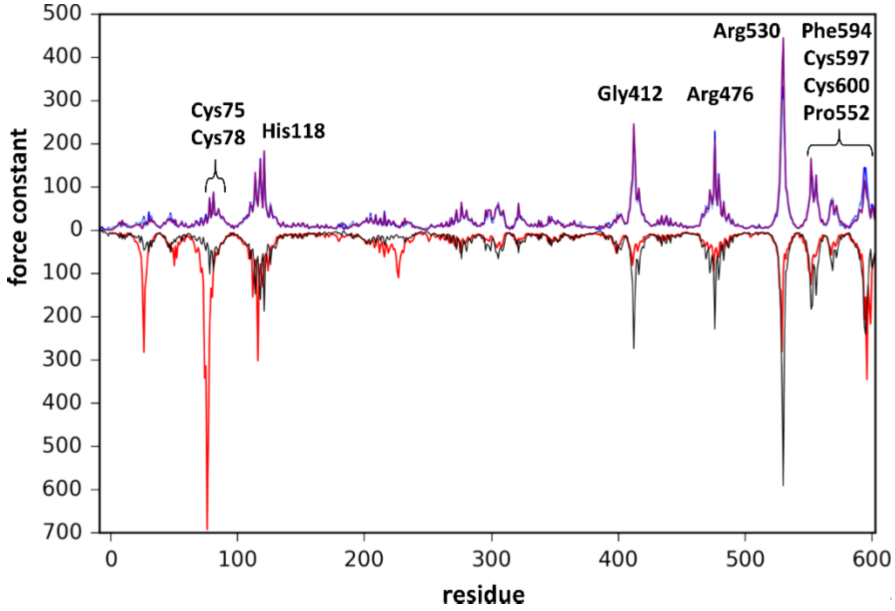

Figure S9. Rigidity profiles of $HoxG_m$ and $HoxG_{MBH}$ and $HoxG_c$. Force constant (in units *kcal·mol*$^{-1}$*·Å*$^{-2}$) plot for time frames taken from the beginning (t = 0 ns), after 100 ns and after 200 ns from GaMD simulation **1**, colored blue, sky-blue and purple. The force constant plots for $HoxG_{MBH}$ and $HoxG_c$ are colored red and black, respectively.

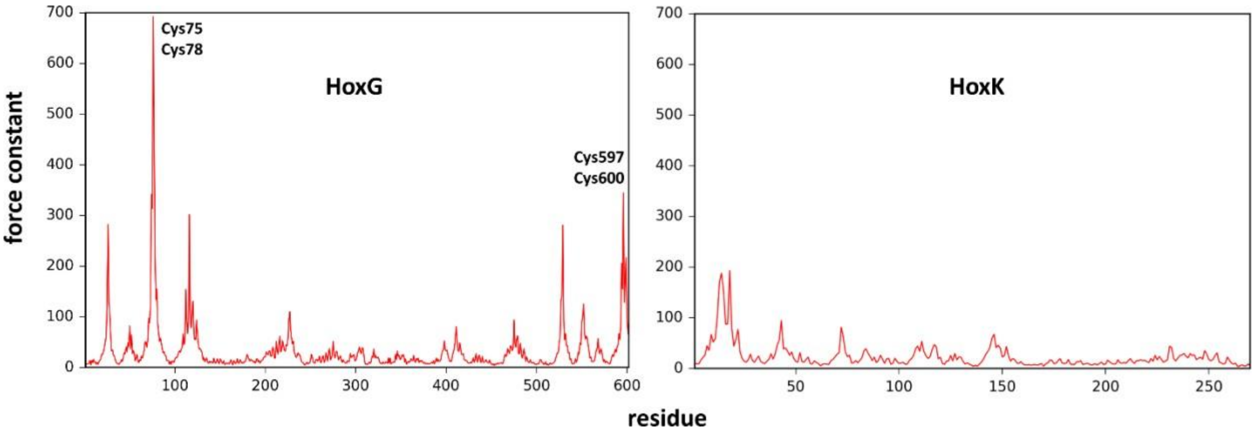

Figure S10. Rigidity profiles of the large subunit HoxG and the small subunit HoxK in the MBH heterodimer from the crystal structure (PDB code: 3RGW [5]. Force constants are expressed in units *kcal·mol*$^{-1}$*·Å*$^{-2}$.

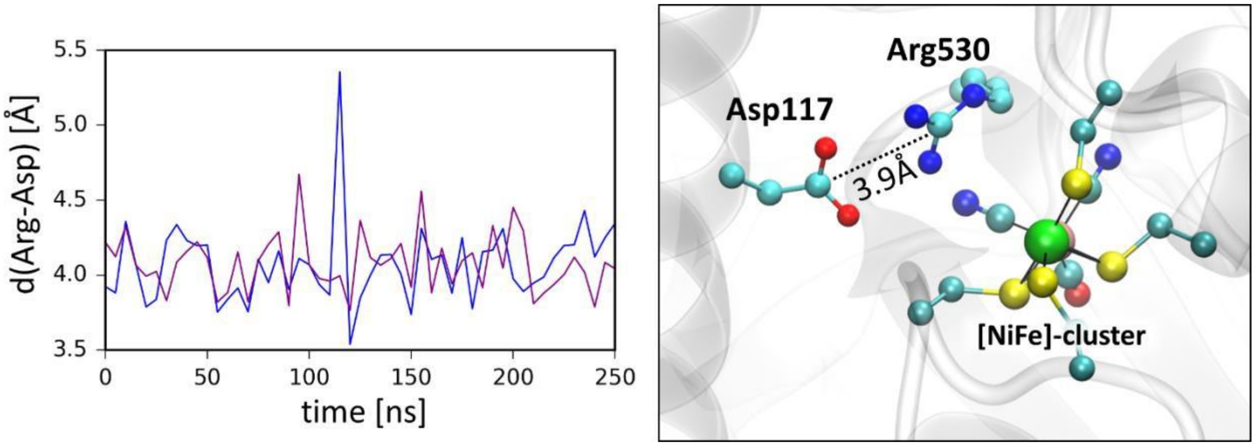

Figure S11. Arg530-Asp117 salt-bridge. **Left:** Time course of distances of the central carbon atom of guanidine group of Arg to the carbon atom of carboxylic group of Asp117 in GaMD simulations **1** (blue) and **2** (purple) of $HoxG_m$. **Right:** Crystal structure (PDB code: 3RGW [5]) conformation.

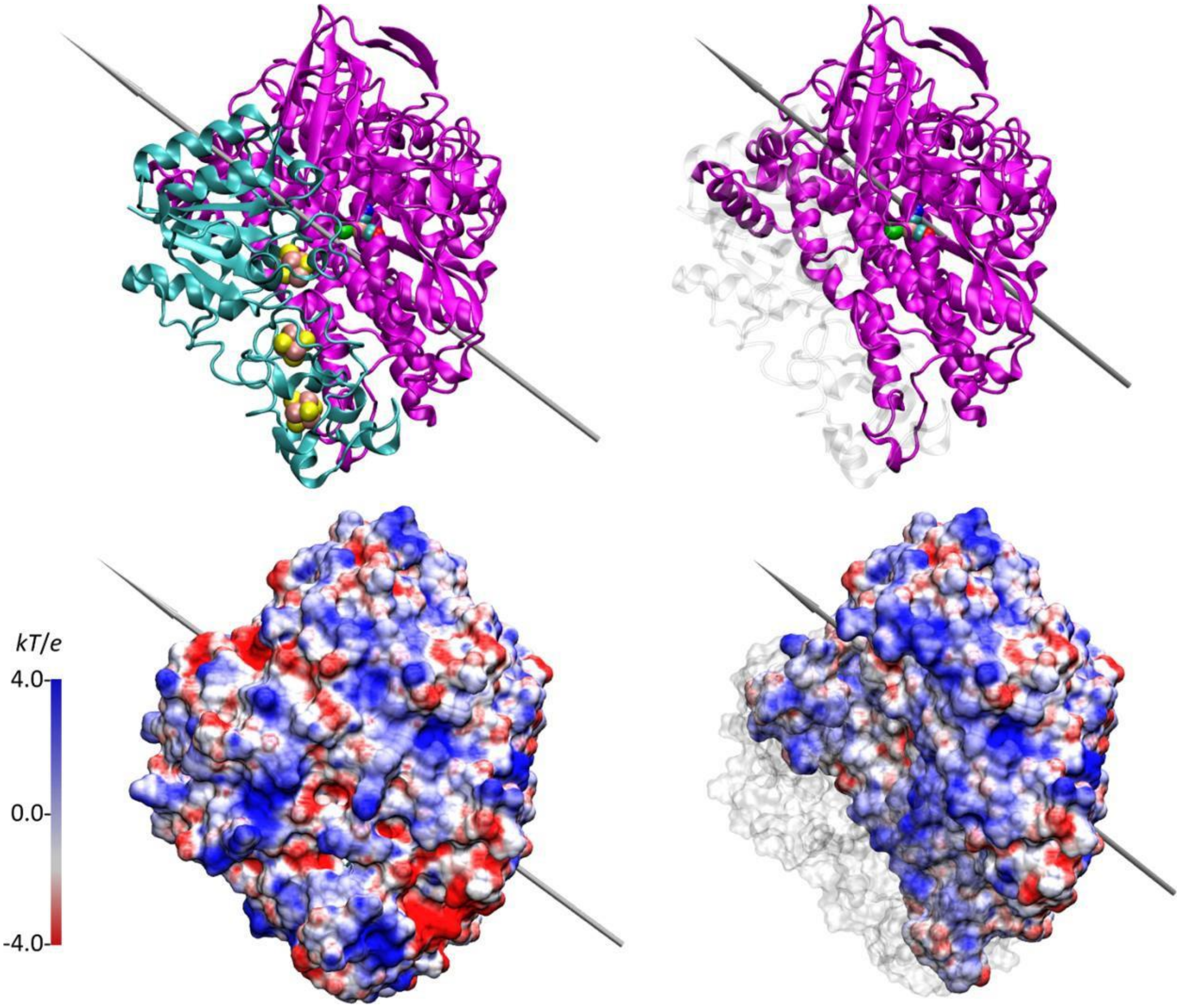

Figure S12. Structure and the electrostatic potential surface of the MBH heterodimer (**Left**) and the respective $HoxG_c$ subunit from the same crystal structure 3RGW [5] (**Right**). The top section of the panel shows secondary structure elements of both systems. The representation and color schemes are taken from Figure 1. The electrostatic potential surface calculated with the APBS [6] (grid resolution 0.3Å) is qualitatively displayed (range: -4 *kT*/*e* to 4 *kT*/*e*), where red and blue indicate negatively and positively charged regions, respectively. The grey arrows indicate the direction of the dipole moments in MBH (ca. 740 Debye) and HoxG (ca. 644 Debye). Charges and radii were used as defined in the CHARMM 36 Force-Field [10,11].

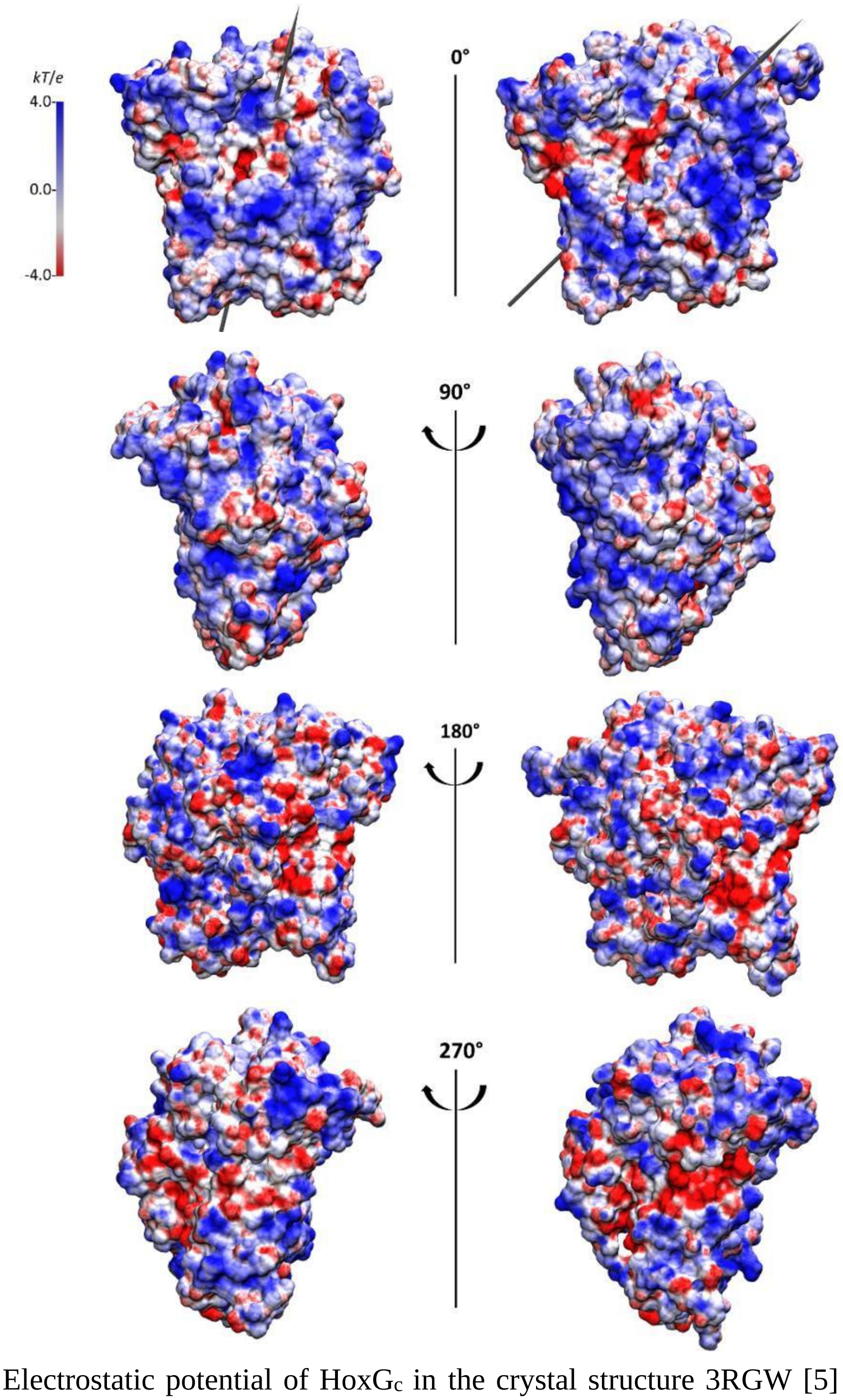

Figure S13: Electrostatic potential of $HoxG_c$ in the crystal structure 3RGW [5] (**left**) and the $HoxG_m$ taken from simulation **2** after 100 ns (**right**). The electrostatic potential surface calculated with the APBS [6] is qualitatively displayed (range: -4 *kT*/*e* to 4 *kT*/*e*) for four orientations of the large subunit. Charges and radii were used as defined in the CHARMM 36 Force-Field [10, 11].

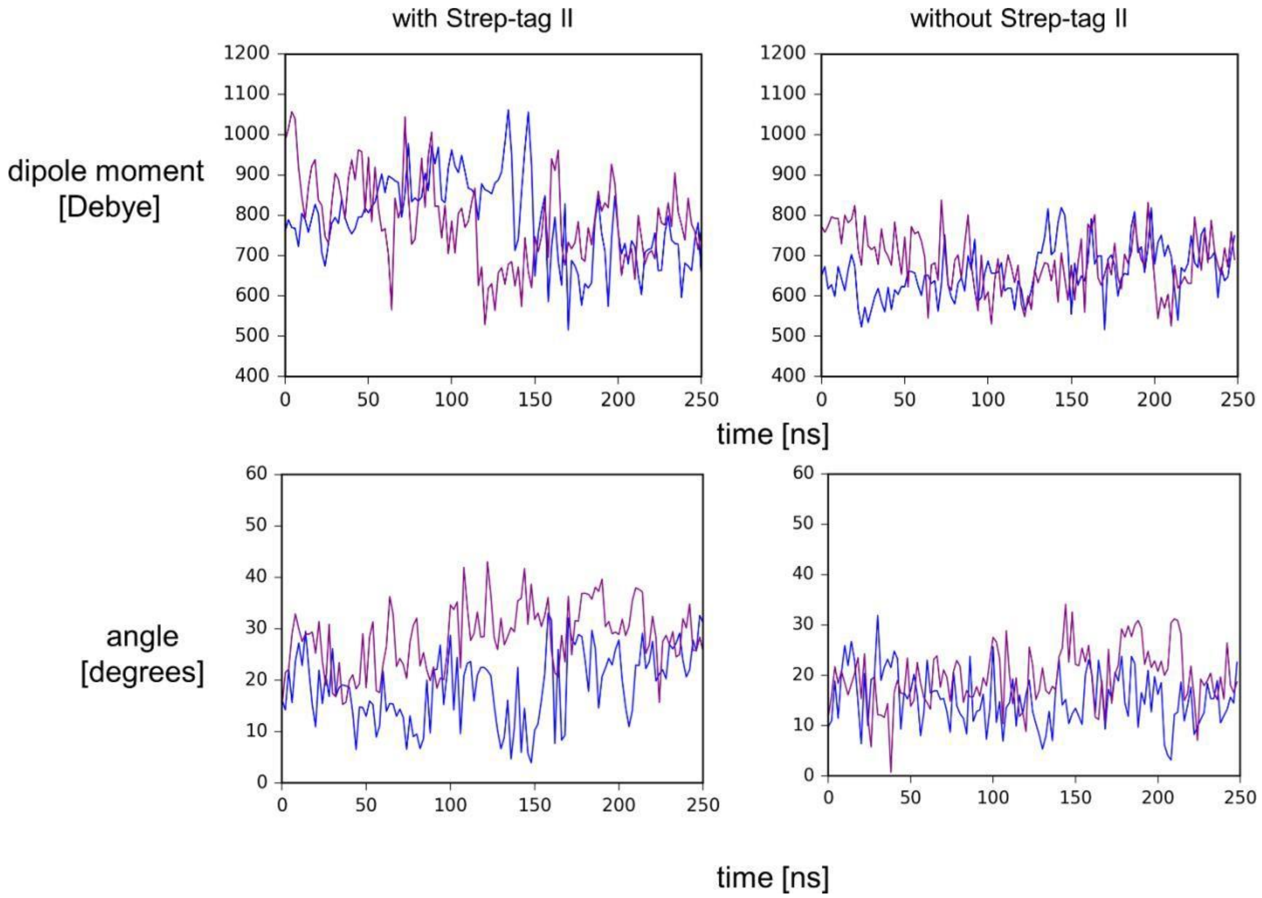

Figure S14. Up: Time course of the intensity of the dipole moment in the $HoxG_m$ in GaMD simulations **1** (blue) and **2** (purple). The blue and purple lines indicate the angle for GaMD simulations 1 and 2, respectively. **Down:** Time course of the angle between the computed dipole moment and that in the initial modelled structure ($HoxG_{MBH}$ in the crystal structure 3RGW [5]).
The blue and purple curves indicate the angle for GaMD simulations **1** and **2**, respectively.

References

[1] Y. Miao, V. A. Feher, and J. A. McCammon, "Gaussian Accelerated Molecular Dynamics: Unconstrained Enhanced Sampling and Free Energy Calculation," *J. Chem. Theory Comput.*, vol. 11, no. 8, pp. 3584–3595, 2015, doi: 10.1021/acs.jctc.5b00436.

[2] T. Meyer and E.-W. Knapp, "p K a Values in Proteins Determined by Electrostatics Applied to Molecular Dynamics Trajectories," *J. Chem. Theory Comput.*, vol. 11, no. 6, pp. 2827–2840, Jun. 2015, doi: 10.1021/acs.jctc.5b00123.

[3] G. Kieseritzky and E. W. Knapp, "Optimizing pKA computation in proteins with pH adapted conformations," *Proteins Struct. Funct. Genet.*, vol. 71, no. 3, pp. 1335–1348, 2008, doi: 10.1002/prot.21820.

[4] B. Rabenstein and E.-W. Knapp, "Calculated pH-Dependent Population and Protonation of Carbon-Monoxy-Myoglobin Conformers," *Biophys. J.*, vol. 80, no. 3, pp. 1141–1150, 2001, doi: 10.1016/S0006-3495(01)76091-2.

[5] J. Fritsch *et al.*, "The crystal structure of an oxygen-tolerant hydrogenase uncovers a novel iron-sulphur centre," *Nature*, vol. 479, no. 7372, pp. 249–253, 2011, doi: 10.1038/nature10505.

[6] N. A. Baker, D. Sept, S. Joseph, M. J. Holst, and J. A. McCammon, "Electrostatics of nanosystems: application to microtubules and the ribosome.," *Proc. Natl. Acad. Sci. U. S. A.*, vol. 98, no. 18, pp. 10037–41, 2001, doi: 10.1073/pnas.181342398.

[7] D. Schneidman-Duhovny, Y. Inbar, R. Nussinov, and H. J. Wolfson, "Geometry-based flexible and symmetric protein docking," *Proteins Struct. Funct. Genet.*, vol. 60, no. 2, pp. 224–231, 2005, doi: 10.1002/prot.20562.

[8] D. Schneidman-Duhovny, Y. Inbar, R. Nussinov, and H. J. Wolfson, "PatchDock and SymmDock: Servers for rigid and symmetric docking," *Nucleic Acids Res.*, vol. 33, no. SUPPL. 2, pp. 363–367, 2005, doi: 10.1093/nar/gki481.

[9] R. A. Laskowski, "PDBsum new things," *Nucleic Acids Res.*, vol. 37, no. SUPPL. 1, pp. 355–359, 2009, doi: 10.1093/nar/gkn860.

[10] R. B. Best *et al.*, "Optimization of the additive CHARMM all-atom protein force field targeting improved sampling of the backbone φ, ψ and side-chain χ1 and χ2 Dihedral Angles," *J. Chem. Theory Comput.*, vol. 8, no. 9, pp. 3257–3273, Sep. 2012, doi: 10.1021/ct300400x.

[11] A. D. MacKerell *et al.*, "All-Atom Empirical Potential for Molecular Modeling and Dynamics Studies of Proteins," *J. Phys. Chem. B*, vol. 102, no. 18, pp. 3586–3616, 1998, doi: 10.1021/jp973084f.

221
